# A top-down strategy for amorphization of hydroxyl compounds for electrocatalytic oxygen evolution

**DOI:** 10.1038/s41467-022-28888-3

**Published:** 2022-03-04

**Authors:** Shangheng Liu, Shize Geng, Ling Li, Ying Zhang, Guomian Ren, Bolong Huang, Zhiwei Hu, Jyh-Fu Lee, Yu-Hong Lai, Ying-Hao Chu, Yong Xu, Qi Shao, Xiaoqing Huang

**Affiliations:** 1 State Key Laboratory of Physical Chemistry of Solid Surfaces, College of Chemistry and Chemical Engineering, Xiamen University, 361005 Xiamen, China; 2 College of Chemistry, Chemical Engineering and Materials Science, Soochow University, 215123 Jiangsu, China; 3 Guangzhou Key Laboratory of Low-Dimensional Materials and Energy Storage Devices, Collaborative Innovation Center of Advanced Energy Materials, School of Materials and Energy, Guangdong University of Technology, 510006 Guangzhou, China; 4 Department of Applied Biology and Chemical Technology, The Hong Kong Polytechnic University, Hung Hom, Kowloon, Hong Kong SAR China; 5 Max Planck Institute for Chemical Physics of Solids, Nothnitzer Strasse 40, 01187 Dresden, Germany; 6 National Synchrotron Radiation Research Center, 101 Hsin-Ann Road, 30076 Hsinchu, Taiwan; 7 Department of Materials Science and Engineering, National Yang Ming Chiao Tung University, 30010 Hsinchu, Taiwan

**Keywords:** Electrocatalysis, Nanoparticles, Materials chemistry

## Abstract

Amorphous materials have attracted increasing attention in diverse fields due to their unique properties, yet their controllable fabrications still remain great challenges. Here, we demonstrate a top-down strategy for the fabrications of amorphous oxides through the amorphization of hydroxides. The versatility of this strategy has been validated by the amorphizations of unitary, binary and ternary hydroxides. Detailed characterizations indicate that the amorphization process is realized by the variation of coordination environment during thermal treatment, where the M–OH octahedral structure in hydroxides evolves to M–O tetrahedral structure in amorphous oxides with the disappearance of the M–M coordination. The optimal amorphous oxide (FeCoSn(OH)_6_-300) exhibits superior oxygen evolution reaction (OER) activity in alkaline media, where the turnover frequency (TOF) value is 39.4 times higher than that of FeCoSn(OH)_6_. Moreover, the enhanced OER performance and the amorphization process are investigated with density functional theory (DFT) and molecule dynamics (MD) simulations. The reported top-down fabrication strategy for fabricating amorphous oxides, may further promote fundamental research into and practical applications of amorphous materials for catalysis.

## Abstract (web summary)

A versatile top-down strategy has been shown to produce amorphous oxides through amorphization of corresponding hydroxides. This approach was used to generate various unitary, binary and ternary amorphous oxides for electrocatalytic oxygen evolution.

## Introduction

Amorphous materials have attracted great attention due to their disordered atomic arrangement and unsaturated coordination environment^1,2^, which have been widely used in diverse fields including mechanical engineering, catalysis, and magnetic applications^3–5^. Compared to their crystalline analogues, amorphous materials consist of continuous random networks instead of periodic structures, and usually display some unique properties^6–8^. Over the past decades, substantial efforts have been devoted to the fabrications and applications of amorphous materials^9,10^. For example, Shao and co-workers reported that the amorphous Ba_0.5_Sr_0.5_Co_0.8_Fe_0.2_O_3−δ_ nanofilms with tunable oxidation state enable up to 315-fold enhanced mass-specific activity towards oxygen evolution reaction (OER) compared to the crystalline BSCF^11^. Huang et al. demonstrated that the amorphization of RuTe_2_ resulted in the local distortion-strain effect, which could abnormally sensitize the Te-pπ coupling capability and enhance the electron transfer of Ru-sites, as a result of significant enhancement on OER performance^12^. Chen and co-workers demonstrated that a transformation of perovskite oxides (ABO_3_) to amorphous motifs via the leaching of A sites or B sites can significantly promote the OER process^13^. Additionally, Zhang et al. demonstrated the lithiation-induced amorphization of layered crystalline Pd_3_P_2_S_8_ can activate this otherwise electrochemically inert material into a highly efficient catalyst for hydrogen evolution reaction (HER)^14^. Therefore, the development of amorphous nanomaterials is of great importance in material science.

Despite these unique properties of amorphous materials, the wide-scale applications of amorphous materials still remain great challenges due to the following reasons: (1) it is still lack of facile protocols for fabricating amorphous materials; (2) High temperature and pressure may further result in recrystallization of amorphous materials, and thus the stability of amorphous materials is strongly limited by the working conditions^15,16^. Therefore, amorphous materials are usually synthesized and used under mild conditions (e.g., low temperature)^17–19^, which severely limits their practical applications. Under such circumstances, pressure-induction and high-temperature cooling strategies have been developed for the formation of amorphous materials, yet suffer from the drawbacks of complicated operation and high cost^20,21^. It thus highly desired to develop facile and versatile strategies for the fabrications of amorphous materials.

Inspired by the previous report that the leaching of A sites or B sites from perovskite oxides (ABO_3_) can lead to the formation of amorphous motifs^13^, we speculate that the structural transformation may lead to the random rearrangement of atoms and thus the formation of amorphous materials. Therefore, we selected CoSn(OH)_6_, a perovskite hydroxide with poor thermal stability which can readily suffer from dehydration at high temperature^22,23^, as a model to systematically study the transformation from highly ordered structure to amorphous oxide. Experimental observations indicate that CoSn(OH)_6_ can be converted into amorphous CoSnO_x_ oxide via a facile low-temperature heat treatment process. Detailed characterizations reveal that the amorphization process experiences the transformation of M–OH octahedron in CoSn(OH)_6_ into M–O tetrahedron in amorphous oxide. Moreover, such strategy can be extended to the fabrications of other binary amorphous oxides (e.g., MgSnO_x_, CaSnO_x_, MnSnO_x_, FeSnO_x_, ZnSnO_x_, and CdSnO_x_) and ternary oxides (e.g., MgCoSnO_x_, CaCoSnO_x_, MnCoSnO_x_, FeCoSnO_x_, NiCoSnO_x_, CuCoSnO_x_, CdCoSnO_x_, and ZnCoSnO_x_), being a versatile strategy for the formation of amorphous materials. The optimal amorphous oxide (e.g., FeCoSn(OH)_6_-300) exhibits promising oxygen evolution reaction (OER) performance in terms of high activity and stability in alkaline media. This work provides a versatile top-down strategy for fabricating amorphous oxides, which may further promote the fundamental researches and practical applications of amorphous materials for catalysis.

## Results

### Synthesis and characterization

**Fig. 1:**
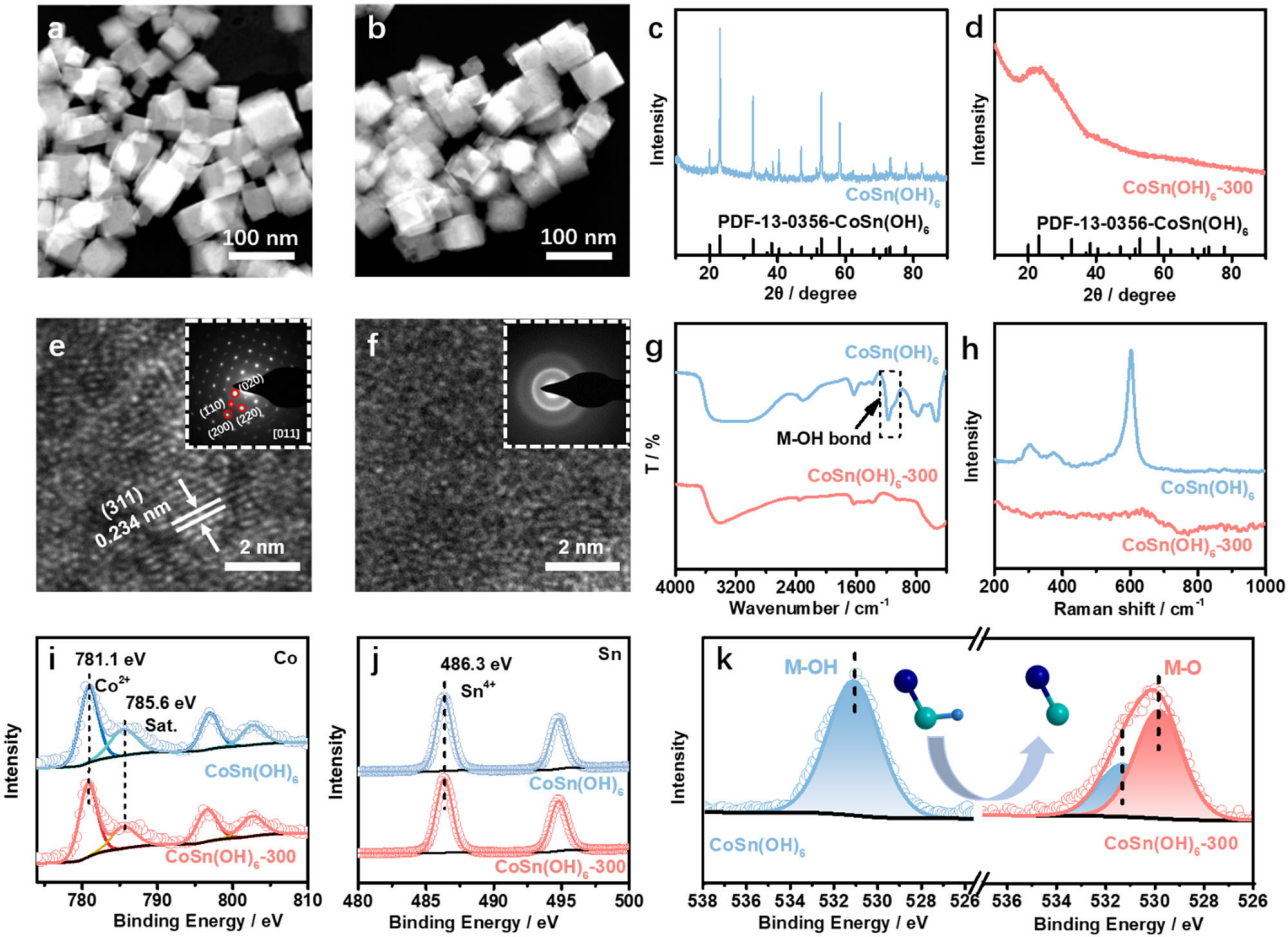
Structural analysis of CoSn(OH)_6_ before and after amorphization. HAADF-STEM images of **a** CoSn(OH)_6_ and **b** CoSn(OH)_6_-300. XRD patterns of **c** CoSn(OH)_6_, and **d** CoSn(OH)_6_-300. HRTEM images of **e** CoSn(OH)_6_ and **f** CoSn(OH)_6_-300. Inset of **e** and **f** are the corresponding SAED patterns. **g** FT-IR and **h** Raman spectra of CoSn(OH)_6_ and CoSn(OH)_6_-300. **i** Co 2*p*, **j** Sn 3*d*, and **k** O 1 *s* XPS spectra of CoSn(OH)_6_ and CoSn(OH)_6_-300.

CoSn(OH)_6_ was synthesized via a precipitation method at room temperature (see details in experimental section). High-angle annular dark-field scanning transmission electron microscopy (HAADF-STEM) image shows that the obtained CoSn(OH)_6_ perovskite hydroxide has a morphology of cube with a mean size is ~50 nm (Fig. 1a). The energy-disperse X-ray spectroscopy (EDS) profile implies that the Co:Sn is close to 1 (Supplementary Fig. 1). After thermal treatment at 300 °C (named as CoSn(OH)_6_-300), no obvious variations in the morphology and size are observed (Fig. 1b). The characteristic peaks in the X-ray diffraction (XRD) pattern are ascribed to CoSn(OH)_6_ perovskite hydroxide (PDF: 13–0356) (Fig. 1c). By sharp contrast, the absence of peaks in the XRD pattern suggests that CoSn(OH)_6_-300 is amorphous (Fig. 1d). High-resolution TEM (HRTEM) image further confirms the transformation from highly crystalline to amorphous structure (Fig. 1e, f), as evidenced by the evolutions of diffraction patterns (inset of Fig. 1e, f). The as-prepared CoSn(OH)_6_ with cubic structure (space group Pn_3_m/224) is confirmed by the selected area electron diffraction (SAED) pattern along the [011] zone axis (inset of Fig. 1e), and the diffraction dots of the (200), (220) and (020) planes are observed. By contrast, the corresponding diffraction halo in SAED pattern confirms the amorphous properties of the materials. Moreover, infrared (IR) and Raman spectroscopy were employed to reveal the amorphization process of CoSn(OH)_6_. As shown in Fig. 1g, the characteristic peak of M–OH bond in CoSn(OH)_6_ at 1200 cm^−1^ disappears after thermal treatment, which might be attributed to the conversion of M–OH bond to M–O bond^24^. Similar phenomena are observed in the Raman spectra of the CoSn(OH)_6_ and CoSn(OH)_6_-300. The four bands at 304–433 and 602 cm^−1^ in the Raman spectrum of CoSn(OH)_6_ are attributed to the breathing vibration of the long M–OH bonds and the bending mode of M–OH–M (bridging OH group), respectively^25^. After amorphization, a broad band appears at 500–750 cm^−1^ in the Raman spectrum of CoSn(OH)_6_-300, which may be attributed to the distorted structure of CoSn(OH)_6_-300 after the transformation from M–O octahedron to M–O tetrahedron^26^. In addition, X-ray photoelectron spectroscopy (XPS) measurement was conducted to investigate the surface properties of CoSn(OH)_6_ and CoSn(OH)_6_-300. No obvious changes are observed in the Co 2*p* and Sn 3*d* XPS spectra^27,28^. The peaks at 781.1 and 785.6 eV in the of Co 2*p* XPS spectra are ascribed to Co^2+^ and the satellite, respectively. The similar features in the XPS spectra of CoSn(OH)_6_ and CoSn(OH)_6_-300 imply the valence states of Co are similar before and after amorphization (i.e., Co^2+^)^29^. Compared to CoSn(OH)_6_, the peak position in the O 1 *s* XPS spectrum of CoSn(OH)_6_-300 negatively shifts by 1.2 eV, which is attributed to the formation of M–O bond in CoSn(OH)_6_-300 (Fig. 1k)^30^.

### Mechanism studies for amorphization

**Fig. 2:**
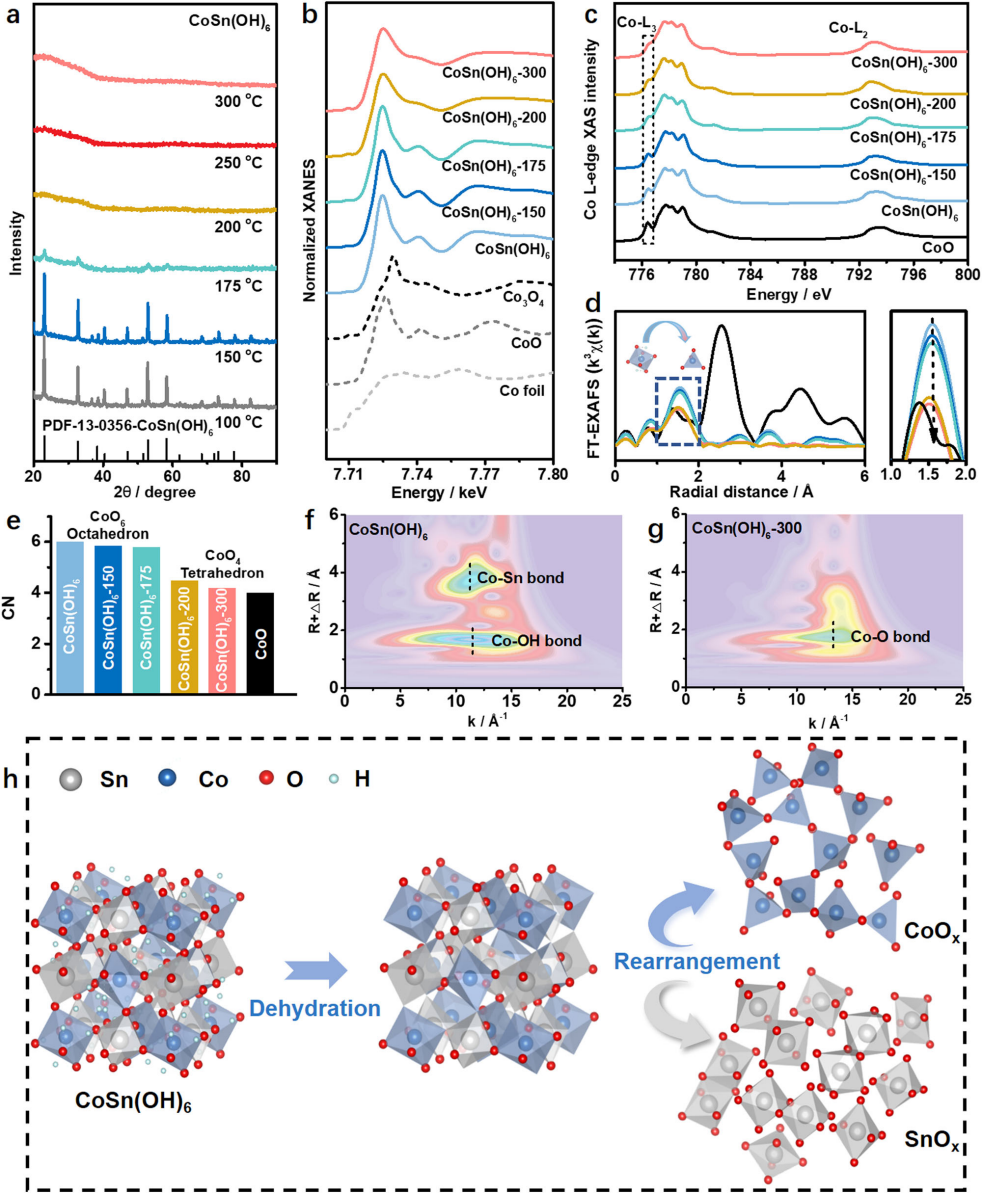
Characterizations for the amorphization. **a** XRD patterns collected after treating CoSn(OH)_6_ at different temperatures. **b** Normalized XANES spectra of various samples at Co-*K* edge. **c** Co-*L*-edge XAS spectra of pristine CoSn(OH)_6_ and CoSn(OH)_6_ treated at different temperatures. **d** Fourier transforms of Co-*K*-edge EXAFS spectra (left) and enlarged spectra at 1–2 Å (right) of different samples. The colours in **d** are same with those in **b**. **e** The average coordination numbers (CNs) of different samples obtained from the fitting of EXAFS spectra. Wavelet transform of Co*-K*-edge EXAFS data of **f** CoSn(OH)_6_ and **g** CoSn(OH)_6_-300. **h** Schematic illustration for the structural evolution of CoSn(OH)_6_ during amorphization.

To understand the process and mechanism of amorphization, thermogravimetric (TG) measurement was performed for CoSn(OH)_6_ in argon (Ar) to study the weight loss during thermal treatment. It is found that CoSn(OH)_6_ experiences a gradual weight loss at 30–240 °C (Supplementary Fig. 2a)^31^. Two intense peaks appear at ~180 and ~240 °C in the derivative thermogravimetric (DTG) curve (Supplementary Fig. 2b), which can be attributed to the dehydration of CoSn(OH)_6_. Moreover, we measured the weight losses by heating CoSn(OH)_6_ to 150 and 250 °C for 1 h with a rate of 10 °C/min (Supplementary Fig. 3). It is found that CoSn(OH)_6_ experiences a gradual weight loss when treated at 150 °C. For CoSn(OH)_6_ treated at 250 °C, it suffers from a rapid weight loss during the heating process, while no obvious weight losses were observed in the next 1 h at the target temperatures, being consistent with above TG analysis. Furthermore, XRD patterns of thermally treated CoSn(OH)_6_ at different temperatures were collected. As shown in Fig. 2a, the characteristic peaks of CoSn(OH)_6_ were reserved in the XRD pattern when CoSn(OH)_6_ were treated at 100 and 150 °C (named as CoSn(OH)_6_-100 and CoSn(OH)_6_-150, respectively). When the temperature was increased to 175 °C (CoSn(OH)_6_-175), the peaks in the XRD patterns are strongly weakened, which completely disappear when the temperature for thermal treatment is over 200 °C, further confirming the amorphization of CoSn(OH)_6_. X-ray absorption near-edge structure spectroscopy (XANES) spectra at the Co-*K* edge were collected to investigate the structures. As shown in Fig. 2b, the Co-*K*-edge XANES spectra of the treated CoSn(OH)_6_ at different temperatures display similar features to that of CoO reference^32–35^, indicating that Co in treated CoSn(OH)_6_ present as Co^2+^. Beisides, the electronic structures were studied by soft X-ray absorption spectroscopy (XAS) at the Co-*L*_*2,3*_ edges, which are highly sensitive to the valence state, spin state environment and local environment^36–40^. Figure 2c shows that the energy position and mutliple spectral features of the Co-*L*-edge XAS spectra of CoSn(OH)_6_. No obvious changes of the valence states of Co (i.e., Co^2+^) are observed during the thermal treatment at different temperatures, whereas the local symmetry changes with the increased temperature for thermal treatment, as revealed by the multiple spectral features (Fig. 2c). The sharp peak at 777.8 eV in the Co-*L*_*3*_ edge of CoO (labelled in Fig. 2c) can be ascribed to Co^2+^ with octahedral coordination. The peak intensity decreases continuously with the increased temperature, suggesting the transformation of Co^2+^ octahedral coordination to tetrahedral coordination^41,42^. In the extended X-ray absorption fine structure (EXAFS) spectra, the peaks at 1.51 and 2.94 Å for CoSn(OH)_6_ are ascribed to Co–OH and Co–Sn coordination, respectively (Fig. 2d)^43^. As increasing the temperature for thermal treatment (over 200 °C), the gradual weakening of Co–OH coordination and the presence of Co–O coordination in the EXAFS spectra of CoSn(OH)_6_-200 and CoSn(OH)_6_-300 suggest the structural evolution. Moreover, the absence of Co–Sn coordination at 2.94 Å suggests the formation of disordered structure^44^. The average coordination number (CN) value derived from the least-squares curve fitting of 1st Co–OH shell was used to reveal the structure of treated CoSn(OH)_6_ (Supplementary Fig. 4)^45,46^. The structural evolutions during the thermal treatment are further validated by the variations of CN (Fig. 2e and Supplementary Table 1). In particular, the slight decrease of CN of Co–OH in the treated samples at 150–175 °C is attributed to the dehydration of CoSn(OH)_6_. After the transformation from Co–OH octahedron to Co–O tetrahedron, the CN of Co–O deceases to 4.2 for CoSn(OH)_6_-300 (Supplementary Table 1). Sepecifically, The CN of CoSn(OH)_6_-300 is similar to that CoO (cubic, F-43m [216]). In addition, wavelet transform (WT) contour plots of CoSn(OH)_6_ and CoSn(OH)_6_-300 have further validated the structural transformation from M–OH to M–O coordination (Fig. 2f, g). Compared to the WT contour plot of CoSn(OH)_6_ with two features of Co–OH to Co–Sn coordination, only one band corresponding to Co–O appears in the WT contour plot of CoSn(OH)_6_-300, indicating the transformation from Co–OH to Co–O coordination in CoSn(OH)_6_-300. Moreover, the absence of Co–Sn coordination further implies the disordered structure of CoSn(OH)_6_-300. Based on the above analysis, we conclude the structural transformation process for CoSn(OH)_6_. Specifically, Co–OH octahedron evolves to Co–O tetrahedron during the decomposition of CoSn(OH)_6_ at high temperature, and the 2nd Co–Sn coordination disappears once Co–O coordination is generated, leading to the formation of disordered structure (Fig. 2h).

**Fig. 3:**
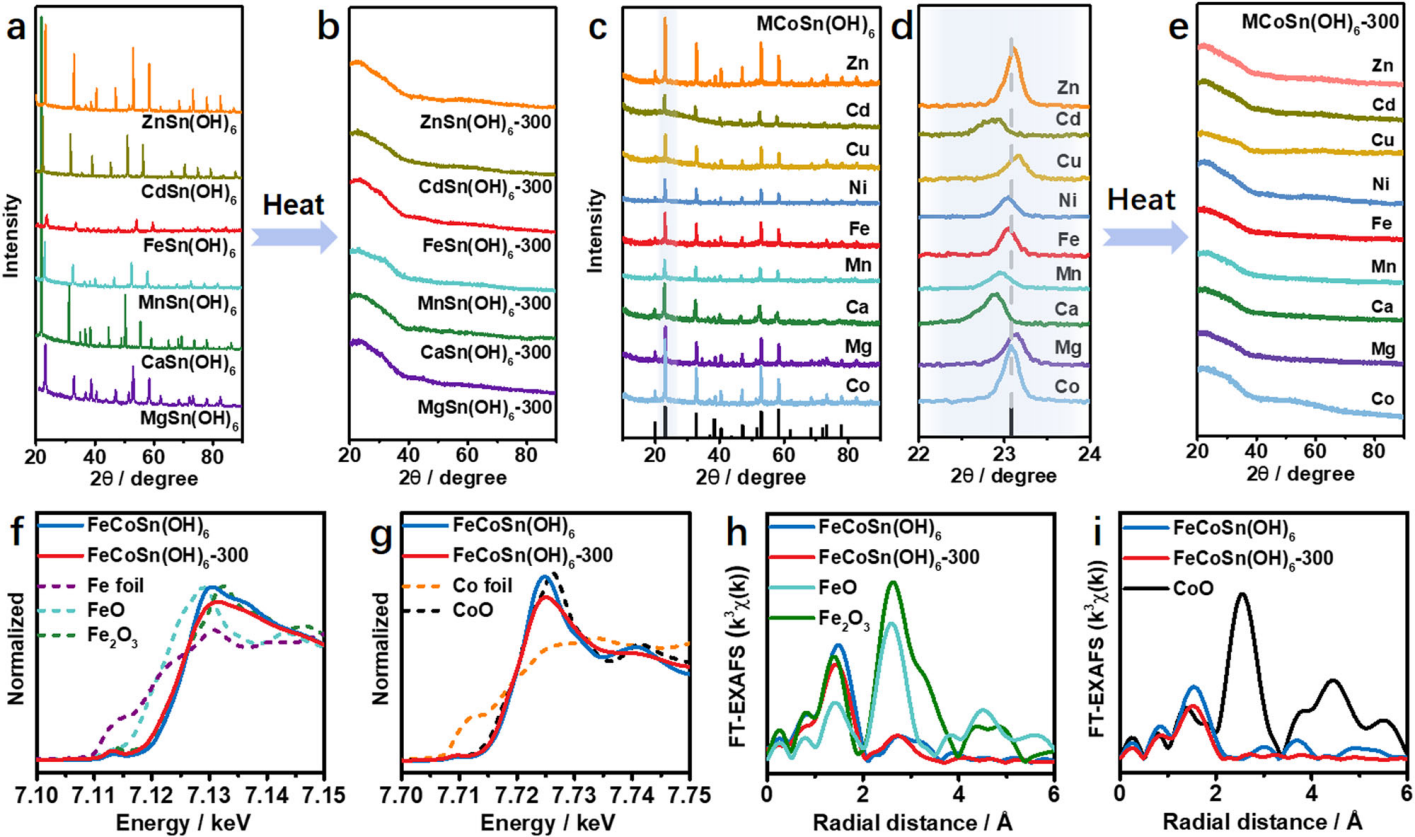
Structural analysis of binary MSn(OH)_6_ and ternary MCoSn(OH)_6_ before and after amorphization. XRD patterns of **a** MSn(OH)_6_, **b** MSn(OH)_6_-300, **c**, **d** MCoSn(OH)_6_, and **e** MCoSn(OH)_6_-300. **f** Normalized Fe *K*-edge XANES spectra of FeCoSn(OH)_6_, FeCoSn(OH)_6_-300, Fe foil, FeO, and Fe_2_O_3_. **g** Normalized Co*-K*-edge XANES spectra of FeCoSn(OH)_6_, FeCoSn(OH)_6_-300, Co foil, and CoO. **h** Fourier transforms of EXAFS spectra of FeCoSn(OH)_6_, FeCoSn(OH)_6_-300, FeO, and Fe_2_O_3_ at Fe*-K* edge. **i** Fourier transforms of EXAFS spectra of FeCoSn(OH)_6_, FeCoSn(OH)_6_-300, and CoO at Co-*K*-edge.

To further study the amorphization of CoSn(OH)_6_, the physicochemical properties of CoSn(OH)_6_-300 were investigated. Compared to the pristine CoSn(OH)_6_, CoSn(OH)_6_-300 displays a much higher surface area of 158 m^2^ g^−1^ (Supplementary Fig. 5), indicating the formation of porous structure after amorphization^47^. It is noted that the amorphous structure is maintained even at a treatment temperature up to 500 °C (Supplementary Fig. 6), suggesting the enhanced thermal stability of the amorphous oxides. Moreover, the morphology, size, and structure of the obtained amorphous structures are largely maintained after scaling up to gram level, showing the great potential of this top-down strategy for the formation of amorphous structures (Supplementary Fig. 7). Furthermore, unitary hydroxide, binary, and ternary perovskite hydroxides were synthesized to demonstrate the versatility of this top-down strategy for the fabrications of amorphous structures. As depicted in Supplementary Figs. 8 and 9, it is found that two-dimensional Co(OH)_2_ and Cd(OH)_2_ nanosheets can be converted into amorphous oxides after thermal treatments in Ar at 400 °C for 1 h. Moreover, various binary perovskite hydroxides including ZnSn(OH)_6_, CdSn(OH)_6_, FeSn(OH)_6_, MnSn(OH)_6_, CaSn(OH)_6_, and MgSn(OH)_6_ were synthesized (Supplementary Fig. 10). With similar manner, these binary perovskite hydroxides experience amorphization to form corresponding binary amorphous oxides (Fig. 3a, b). Besides, the generality of this top-down strategy has been validated by the amorphization of CdIr(OH)_6_, a Sn-free binary hydroxide (Supplementary Fig. 11). Additionally, ternary perovskite hydroxides, such as ZnCoSn(OH)_6_, CdCoSn(OH)_6_, CuCoSn(OH)_6_, NiCoSn(OH)_6_, FeCoSn(OH)_6_, MnCoSn(OH)_6_, CaCoSn(OH)_6_, and MgCoSn(OH)_6_ (Supplementary Fig. 12), can also be transformed into amorphous oxides through this top-down strategy (Fig. 3c–e). We take FeCoSn(OH)_6_ as an example, SEM and TEM images indicate that ternary FeCoSn(OH)_6_ perovskite hydroxide has a spherical shape with a mean size of 100 nm (Supplementary Fig. 13a, b). Elemental mapping image and line scan profile suggest that all the elements are evenly distributed in the nanospheres (Supplementary Fig. 13c, d). The surface area of ternary FeCoSn(OH)_6_ perovskite hydroxide is 62.0 m^2^ g^−1^ (Supplementary Fig. 13e). After thermal treatment, no obvious changes of the morphology, size, and elemental distributions are observed for the amorphous oxide, and the disappearance of lattice fringes in HRTEM image confirms the amorphization of FeCoSn(OH)_6_ (Supplementary Fig. 14a–d). Correspondingly, the surface area significantly increases to 163.6 m^2^ g^−1^ after amorphization (Supplementary Fig. 14e). The Sn 3*d* and Co 2*p* XPS spectra of the amorphous FeCoSn(OH)_6_-300 display similar features to the pristine FeCoSn(OH)_6_, whereas the intensity of M–O in the XPS spectrum of FeCoSn(OH)_6_-300 is much higher than that of FeCoSn(OH)_6_, which is ascribed to FeCoSn(OH)_6_ dehydration during thermal treatment (Supplementary Fig. 15). XANES spectra of FeCoSn(OH)_6_ show that the valence states of Fe and Co are close to Fe^3+^ and Co^2+^, respectively (blue spectra in Fig. 3f, g). Under the treatment in H_2_/Ar (5 vol.%) at 300 °C for 1 h, the chemical valences of Fe^3+^ and Co^2+^ are largely maintained, as revealed by the similar features in Fe *K*-edge and Co-*K*-edge XANES spectra (red spectra in Fig. 3f, g). Besides, the variations of local electronic properties after amorphization have been further validated by the Fourier-transformed EXAFS spectra. As shown in Fig. 3h, i, the presence of Fe–O (Co–O) coordination and the absence of Fe–OH (Co–OH) and 2nd Fe–M (Co–M) coordination in the EXAFS spectra of FeCoSn(OH)_6_-300 confirm the amorphization of FeCoSn(OH)_6_ after the treatment. Detailed analysis indicates that the CN of Fe–OH and Co–OH in FeCoSn(OH)_6_ are 5.0 and 5.6, respectively. After the thermal treatment, the CNs of Fe–O and Co–O in FeCoSn(OH)_6_-300 decrease to 3.9 and 4.4, respectively, suggesting that the octahedral Fe–OH and Co–OH coordination are transformed into tetrahedral Fe–O and Co–O coordination (Supplementary Figs. 16, 17 and Supplementary Table 2). The absence of M–M (M = Fe, Co, and Sn) coordination in the EXAFS spectrum of FeCoSn(OH)_6_-300 further confirms the disordered structure of FeCoSn(OH)_6_-300. In addition, the versatility of such top-down strategy has been validated by fabricating FeCoSn(OH)_6_ with different compositions. As shown in Supplementary Fig. 18, as increasing the Fe/Co ratio in FeCoSn(OH)_6_, all the products display similar XRD patterns, indicating that Fe can occupy the position of Co to form a polynary perovskite hydroxide. Note that the positive peak shifts is attributed to the lattice contraction due to the increase of Fe/Co ratio.

### Catalytic performance of amorphous oxides for OER

**Fig. 4:**
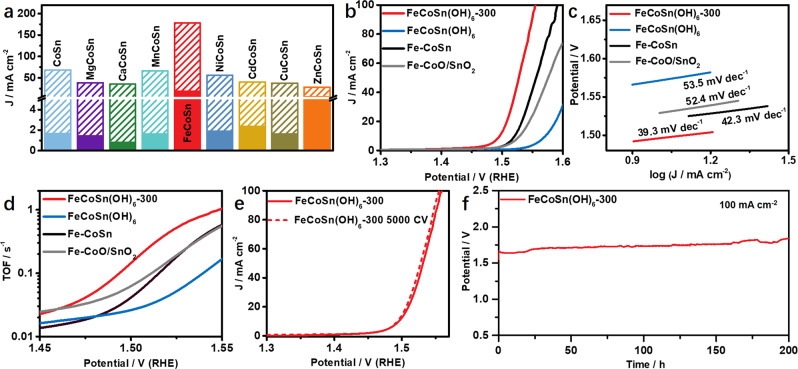
Catalytic performance of OER. **a** The current densities of MCoSn(OH)_6_ and MCoSn(OH)_6_-300 in O_2_-saturated 1.0 M KOH solution. **b** OER polarization curves of FeCoSn(OH)_6_-300, FeCoSn(OH)_6_, Fe-CoSn, and Fe-Co/SnO_2_. **c** Tafel slopes of different catalysts. **d** TOF values collected at different potentials for FeCoSn(OH)_6_-300, FeCoSn(OH)_6_, Fe-CoSn, and Fe-Co/SnO_2_. **e** OER polarization curves of FeCoSn(OH)_6_-300 after 5000 cycles. **f** Continuous OER chronopotentiometry test for FeCoSn(OH)_6_-300 at 100 mA cm^−2^.

To study the catalytic performance of the obtained amorphous oxides, oxygen evolution reaction (OER) was performed in O_2_-saturated 1.0 M KOH solution. It is found that the amorphous FeCoSn(OH)_6_-300 exhibits superior OER performance to other amorphous oxides. Specifically, the current density reaches 178 mA cm^−2^ at 1.6 V (vs. RHE) for FeCoSn(OH)_6_-300, which is significantly higher than those of other amorphous oxides (Fig. 4a and Supplementary Fig. 19). On the other hand, the OER activity of FeCoSn(OH)_6_-300 is 39.4 times higher than that of the FeCoSn(OH)_6_, indicating that the amorphization can significantly improve the OER performance (Fig. 4a). To further demonstrate the significance of amorphization on OER, FeCoSn(OH)_6_ was further converted into crystalline Fe-CoO/SnO_2_ and Fe-CoSn alloy by annealing at 700 °C in Air and H_2_/Ar, respectively (Supplementary Figs. 20 and 21). As shown in Fig. 4b, the overpotentials for FeCoSn(OH)_6_, Fe-CoSn alloy and Fe-CoO/SnO_2_ at 10 mA cm^−2^ are 342, 291, and 299 mV, respectively, which are much higher than that of FeCoSn(OH)_6_-300 (266 mV) under the same conditions. Moreover, the Tafel slope of FeCoSn(OH)_6_-300 is 39.3 mV dec^−1^, which is significantly lower than that of Fe-CoO/SnO_2_ (52.4 mV dec^−1^), Fe-CoSn (42.3 mV dec^−1^), and FeCoSn(OH)_6_ (53.5 mV dec^−1^) (Fig. 4c). Results from electrochemical active surface area (ECSA) measurement indicate that the amorphous FeCoSn(OH)_6_-300 exhibits superior OER activity to FeCoSn(OH)_6_, Fe-CoSn, Fe-CoO/SnO_2_, and FeCoSn(OH)_6_ (Supplementary Figs. 22 and 23)^48^. Nyquist plots imply that FeCoSn(OH)_6_-300 has a smaller interface charge-transfer resistance than those of other references (Supplementary Fig. 24)^49^. Besides, we calculated the turnover frequency (TOF) values based on the surface atoms. As shown in Fig. 4d, the TOF value of FeCoSn(OH)_6_-300 at 300 mV is 0.622 s^−1^, which is ~9.7, ~2.6, and ~2.6 times higher than that of FeCoSn(OH)_6_, Fe-CoSn alloy, and Fe-CoO/SnO_2_, respectively. Additionally, cyclic voltammograms (CV) was used to evaluate the stability of FeCoSn(OH)_6_-300 for OER, where FeCoSn(OH)_6_-300 exhibits promising stability without decrease of OER activity after 5000 cycles (Fig. 4e). No obvious increase of OER potential during chronopotentiometric (CP) experiment at 100 mA cm^−2^ for 200 h indicates the excellent stability of FeCoSn(OH)_6_-300 (Fig. 4f). The morphology and amorphous structure of the spent FeCoSn(OH)_6_-300 catalyst are largely maintained after OER test (Supplementary Fig. 25a–c). Note that the dissolution of Sn can lead to the formation of defects and enhance the catalytic performance (Supplementary Fig. 25d)^50^. It is found that Co on the surface of amorphous FeCoSn(OH)_6_-300 is slightly oxidized into CoOOH after activation and stability test (Supplementary Fig. 26), which agrees with the previous reports^29,51^. The above results suggest that the amorphization FeCoSn(OH)_6_ can significantly enhance the OER performance (Supplementary Table 3).

### Theoretical calculation of reaction process and amorphous process

**Fig. 5:**
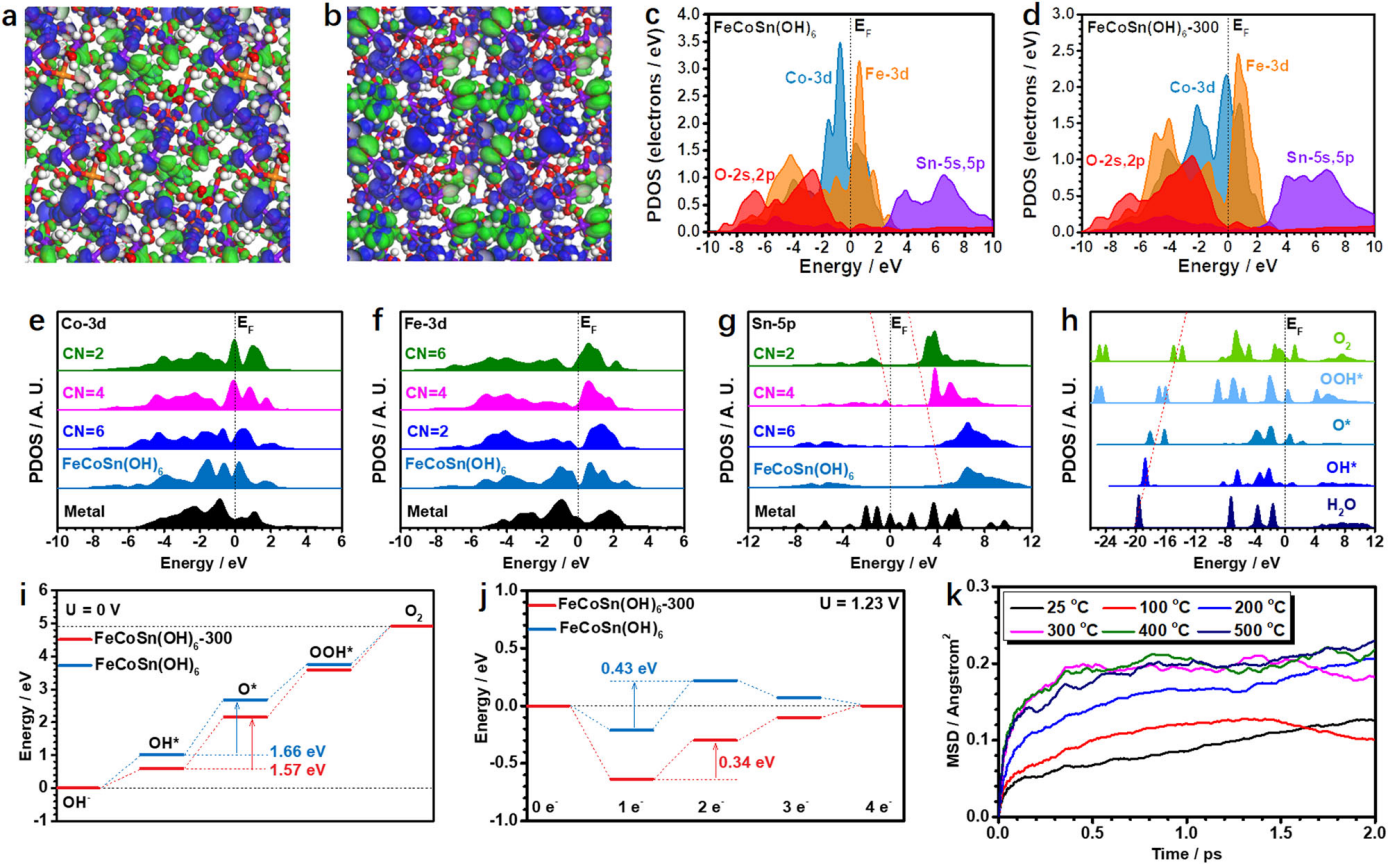
DFT calculations of reaction process and amorphous process. The 3D contour plot of electronic distribution near Fermi level of **a** FeCoSn(OH)_6_ and **b** FeCoSn(OH)_6_-300. Blue balls: Co, orange balls: Fe, purple balls: Sn, red ball: O, white balls: H. Blue isosurface: bonding orbitals, and green isosurface: antibonding orbitals. The PDOS of **c** FeCoSn(OH)_6_ and **d** FeCoSn(OH)_6_-300. **e** The site-dependent PDOS of **e** Co sites, **f** Fe sites, and **g** Sn-5p sites. **h** PDOS of key adsorbates in OER on amorphous FeCoSn(OH)_6_-300. **i** The energetic trend of OER at U = 0 V. **j** The energetic trend of OER at U = 1.23 V. **k** The mean square displacements (MSD) of FeCoSn(OH)_6_ at different temperatures.

To understand the superior OER performance of the amorphous FeCoSn(OH)_6_, density functional theory (DFT) calculations were performed to study the electronic modulations and the reaction trends (Supplementary Fig. 27, see more discussion in Methods section). With respect to FeCoSn(OH)_6_, it is noted that the surface Co sites with unsaturated coordination mainly contribute to the bonding orbitals, while the Co sites and OH groups mainly contribute to the antibonding orbitals (Fig. 5a). The contributions of Sn sites are limited in both bonding and antibonding orbitals near the Fermi level (E_F_). After amorphization, the surface of FeCoSn(OH)_6_-300 is electron-rich due to the presences of low-coordinated Co and Fe sites (Fig. 5b). In this case, the antibonding orbitals are dominated by the OH groups. Moreover, the orbital coupling of the surface OH groups may facilitate the electron transfer. The projected partial density of states (PDOS) have further proved the electronic modulations (Fig. 5c). For FeCoSn(OH)_6_, Co-3d shows a sharp peak at E_V_–0.73 eV (E_V_ = 0 eV) with the e_g_-t_2g_ splitting of 1.12 eV. Fe-3d orbitals suggest the significant contribution of the electron density near E_F_ with the e_g_-t_2g_ splitting of 1.69 eV. The Sn-5p orbitals locate above the E_F_ while the O-2s and 2p orbitals show the dominant peak at E_V_–2.83 eV, and therefore O can serve as the electron reservoir. For FeCoSn(OH)_6_-300, the Co-3d orbitals upshift towards the E_F_ (−0.11 eV), and the e_g_-t_2g_ splitting of Fe-3d significantly increases to 1.98 eV (Fig. 5d). Given that the dominant peak of Co-3d orbitals in the middle of e_g_-t_2g_ orbitals of Fe-3d and the upshift of O-2s and 2p orbitals (E_V_–2.42 eV), as well as the similar Sn-5p orbitals after the amorphization compared with FeCoSn(OH)_6_, the p-d couplings between Co and Fe sites may be strongly promoted. Moreover, we investigated the site-dependent electronic structures for the FeCoSn(OH)_6_-300 (Fig. 5e). For Co-3d sites, the decrease of coordination numbers (CN) will lead to the increased electron density near the E_F_ and thus promotes the electroactivity towards OER. In contrast, Fe-3d orbitals deliver an opposite dependence of the electronic structures with respect to CN (Fig. 5f). The e_g_-t_2g_ splitting becomes larger with the increased CN, indicating that the pinning effect may play a critical role to stabilize the valence states of Co active sites. For Sn sites, it is revealed that the decrease of CN leads to the downshift of 5p orbitals towards E_F_ and the decrease of energy barriers for electron transfer (Fig. 5g). For the key intermediates, the O-species from H_2_O^*^ to O_2_ show the linear correlation of the σ components in 2p orbitals, which guarantees the efficient conversion of intermediates to O_2_ during OER (Fig. 5h). Furthermore, the reaction trend of OER has been compared for the amorphous FeCoSn(OH)_6_-300 and crystallized FeCoSn(OH)_6_. Owing to the strong binding strength of OH^*^ on FeCoSn(OH)_6_-300 and FeCoSn(OH)_6_, the energy barriers for the conversion from OH^*^ to O^*^ (rate-determining step) are 1.57 and 1.66 eV, respectively (Fig. 5i). Further calculations imply that OH^*^ can spontaneously adsorb on the surfaces of FeCoSn(OH)_6_-300 and FeCoSn(OH)_6_ when the equilibrium potential (U = 1.23 V) was applied (Fig. 5j). The energy barrier for the conversion of OH^*^ to O^*^ is 0.34 eV for FeCoSn(OH)_6_-300, which is smaller than that for FeCoSn(OH)_6_ (0.43 eV), further confirming the enhanced electroactivity for OER over FeCoSn(OH)_6_-300. Additionally, we have carried out the molecule dynamic (MD) simulations under temperatures from 298 to 773 K to understand the amorphization of CoSn(OH)_6_. As shown in Fig. 5k, the mean square displacement (MSD) results show that the overall atomic movements are limited at the temperature range of 298–373 K. When the temperature was increased to 473 K, the significant increase of MSD value suggests the initiation of amorphization. No obvious increase of the MSD value is observed when the temperature is increased from 573 to 773 K, suggesting the completion of amorphization. MD results suggest the amorphization occurs at the temperature range of 473–573 K, being consistent with experimental observation.

## Discussion

In summary, we have demonstrated a versatile top-down strategy for the fabrications of amorphous oxides via a low-temperature thermal treatment of perovskite hydroxide. The generality of this strategy has been validated by the amorphizations of various unitary hydroxides, binary, and ternary perovskite hydroxides. Detailed characterizations indicate that the amorphization process is realized by the variations of coordination environment during thermal treatment. Specifically, the octahedral M–OH coordination in perovskite hydroxide evolves to tetrahedral M–O coordination in amorphous oxides with the disappearance of the 2nd M–M during the amorphization process. Benefiting from the disordered structure and the regulated electronic properties of amorphous oxides, the ternary amorphous oxide of FeCoSn(OH)_6_-300 can sever as promising catalyst for alkaline OER with superior activity and stability to many reported catalysts. The obtained amorphous oxides can also exhibit excellent stability without crystallization even when the temperature is as high as 500 °C. DFT calculations imply that the amorphization can strongly promote the p-d couplings between Co and Fe sites, which significantly decrease the energy barrier for the conversion from OH^*^ to O^*^, the rate-determining step for OER. Moreover, MD calculations confirm that the amorphization occurs at the temperature range of 473–573 K, being consistent with experimental observation. This work not only provides a robust strategy for fabricating various amorphous oxides but also may shed new light on nanomaterials, catalysis, chemistry, and beyond.

## Methods

### Chemicals

Magnesium chloride hexahydrate (MgCl_2_·6H_2_O, 98%), calcium chloride (CaCl_2_, 96%), manganese chloride tetrahydrate (MnCl_2_·4H_2_O, 99%), zinc chloride (ZnCl_2_, 98%), cobalt chloride hexahydrate (CoCl_2_·6H_2_O, 99%), nickel chloride hexahydrate (NiCl_2_·6H_2_O, 98%), cadmium chloride (CdCl_2_, 99%), copper chloride dihydrate (CuCl_2_·2H_2_O, 98%), stannic chloride pentahydrate (SnCl_4_·5H_2_O, 98%), 2-aminoethanol (HO(CH_2_)_2_NH_2_, 99.9%), potassium hydroxide (KOH, 85%), and ethanol (C_2_H_5_OH, 99.7%) were obtained from Sinopharm Chemical Reagent Co. Ltd. Hydrogen hexachloroiridate (IV) hexahydrate (H_2_IrCl_6_·H_2_O, 99%) was obtained from Alfa Aesar. Ferrous chloride tetrahydrate (FeCl_2_·4H_2_O) was obtained from Strem. Nafion solution (~5 wt.% in a mixture of lower aliphatic alcohols and water) was obtained from Sigma–Aldrich. The carbon powder (XC72R) was purchased from Vulcan. The deionized water (18 MΩ cm^−1^) used in all experiments was prepared by passing water through an ultrapure purification system.

### Syntheses of M(OH)_2_ and M(OH)_2_-400

0.2 mmol MCl_2_ (M = Co and Cd) was dissolved in 15 mL KOH (0.1 M) solution under ultrasonication and then kept undisturbed for 12 h at room temperature. The resulting precipitation was washed with deionized water by three times and dried at 80 °C in an oven for 12 h. The M(OH)_2_-400 was prepared by calcining M(OH)_2_ at 400 °C in H_2_/Ar (5 vol.%) for 1 h.

### Synthesis of binary MSn(OH)_6_ and ternary MCoSn(OH)_6_

51.6 mg SnCl_4_·5H_2_O and 0.2 mmol MCl_2_ (M = Mg, Ca, Mn, Fe, Co, Ni, Cu, Cd, and Zn) was dissolved in 15 mL deionized water, followed by dropwise adding 0.5 mL 2-aminoethanol under ultrasonic treatment for 15 min. Then, the mixture was kept undisturbed at room temperature for 12 h. The resulting precipitation was washed with deionized water by three times and dried at 80 °C in an oven for 12 h. The protocol for synthesizing ternary MCoSn(OH)_6_ perovskite hydroxides is similar to that for binary MSn(OH)_6_ except for changing the amounts of corresponding precursors. The detailed parameters are listed in Supplementary Table 4.

### Synthesis of amorphous MSn(OH)_6_-300 and MCoSn(OH)_6_-300

The MSn(OH)_6_-300 or MCoSn(OH)_6_-300 were prepared by annealing MSn(OH)_6_ or MCoSn(OH)_6_ (M = Mg, Ca, Mn, Fe, Co, Ni, Cu, Cd, and Zn) at 300 °C in H_2_/Ar (5 vol.%) for 1 h.

### Synthesis of Fe-CoO/SnO_2_ and Fe-CoSn

Fe-CoO/SnO_2_ and Fe-CoSn were prepared by annealing FeCoSn(OH)_6_ at 700 °C for 1 h in Air and H_2_/Ar (5 vol.%), respectively.

### Synthesis of CdIr(OH)_6_ and CdIr(OH)_6_-300

20.4 mg H_2_IrCl_6_·H_2_O was dissolved in 10 mL KOH solution (0.1 M), followed by adding 9.15 mg CdCl_2_ under ultrasonication and then kept undisturbed for 12 h at room temperature. The resulting precipitation was washed with deionized water by three times and dried at 80 °C in an oven for 12 h to obtain CdIr(OH)_6_. For the synthesis of CdIr(OH)_6_-300, CdIr(OH)_6_ was calcinated at 300 °C in H_2_/Ar (5 vol.%) for 1 h.

### Characterization

Low-magnification TEM was acquired on a HITACHI HT7700 transmission electron microscope at an accelerating voltage of 120 kV. High-resolution TEM (HRTEM) and high-angle annular dark-field scanning TEM (HAADF-STEM) were conducted on a FEI Tecnai F20 transmission electron microscope at an acceleration voltage of 200 kV. X-ray diffraction (XRD) patterns were collected on a Shimadzu XRD-6000 X-ray diffractometer. Scanning electron microscopy energy-dispersive X-ray spectroscopy (SEM-EDS) spectra were obtained with a HITACHI S-4700 cold field emission scanning electron microscope. Fourier transform infrared spectra were recorded on a VERTEX 70 spectrometer (Bruker). Raman spectra were recorded on a Horiba HR800 Raman spectrometer using the 633 nm laser as the excitation source. X-ray photoelectron spectra were collected with an SSI S-Probe XPS Spectrometer. The carbon peak at 284.6 eV was used as a reference for calibration. XAS data were collected at the TLS17C1 and TLS11A beamline of the National Synchrotron Radiation Research Center (NSRRC, Hsinchu, Taiwan), respectively. Data were processed according to standard procedures using the Demeter program package (Version 0.9.24)^52^.

### Electrochemical measurements

The electrochemical measurements were performed by using a CHI 660E workstation (Chenhua, Shanghai) with a three-electrode configuration. All the experiments were carried out at room temperature. Saturated calomel electrode and Graphite rod were used as the reference and counter electrode, respectively. The inks of different electrocatalysts were prepared by sonicating 2 mg catalyst and 2 mg carbon powder with 0.4 mL isopropanol and 15 μL Nafion for 30 min. The working electrode was then fabricated by dropping 40 μL ink onto a glass carbon electrode (GCE) with a geometric area of 0.196 cm^2^. Linear-sweep voltammograms and chronopotentiometry measurements were carried out to study the catalytic activity and stability, respectively. The Linear-sweep voltammograms measurements were operated on a GCE, and the chronopotentiometry measurements were operated on a carbon paper (0.25 cm^2^). All the polarization curves were 95% iR corrected and the linear scan voltammetry at 5 mV s^−1^ for each sample.

### ECSA calculations

The ECSA was calculated by the method of C_dl_.

1 $${{{{{\rm{ECSA}}}}}}=\frac{{C}_{{{{{{\rm{dl}}}}}}}}{{C}_{{{{{{\rm{s}}}}}}}}$$

where Cs is the specific capacitance of the sample or the capacitance of an atomically smooth planar surface of the material per unit area under identical electrolyte conditions. For our estimates of surface area, we use general specific capacitances of Cs = 0.040 mF cm^−2^ in 1 M KOH based on typical reported values.

### TOF calculations

For OER, the TOF value is usually calculated by the equation:

2 $${{{{{\rm{TOF}}}}}}=\frac{J\times A}{4\times F\times n}$$

where J is the current density after 95% iR corrected, A is the geometric area of the electrode (0.196 cm^2^), F is Faraday’s constant and n is the molar number of active sites. In our study, we suppose Co and Fe as active sites for catalysts.

The surface area of each cobalt oxide:

3 $${{{{{{\rm{S}}}}}}}_{{{{{{\rm{atom}}}}}}}=4.838\times {\left[\frac{11.63}{{6.023\times 10}^{23}}\right]}^{\frac{2}{3}}\,{{{{{{\rm{cm}}}}}}}^{2}{{{{{{\rm{atom}}}}}}}^{-1}$$

The molar number of atoms exposed on the surface:

4 $${{{{{\rm{n}}}}}}=\frac{{{{{{\rm{ECSA}}}}}}}{{3.478\times 10}^{-15}\,{{{{{{\rm{cm}}}}}}}^{2}\,{{{{{{\rm{atom}}}}}}}^{-1}\times {{{{{{\rm{N}}}}}}}_{{{{{{\rm{A}}}}}}}}$$

The number n was estimated via the total loading mass, according to the equation:

5 $${{{{{\rm{n}}}}}}=\frac{{{{{{\rm{m}}}}}}\times {{{{{{\rm{N}}}}}}}_{{{{{{\rm{A}}}}}}}}{{M}_{w}}$$

where m is the loading mass, N_A_ is Avogadro’s constant, and M_w_ is the molecular weight of the catalysts.

### Calculation Setup

DFT calculations were used to investigate the enhanced catalytic performance through the CASTEP packages^53^. For all the calculations, we selected the generalized gradient approximation (GGA) with Perdew-Burke-Ernzerhof (PBE) to supply an accurate description of the exchange-correlation energy^54–56^. The plane-wave basis cutoff energy was set to 380 eV based on the ultrasoft pseudopotentials. For all the geometry optimizations in this work, the Broyden-Fletcher-Goldfarb-Shannon (BFGS) algorithm is selected^57^. The k-points with coarse quality have been applied for all the energy minimizations. For all the geometry optimizations, the calculations have to satisfy the following convergence criteria that the Hellmann-Feynman forces on the atom should not exceed 0.001 eV/Å, and the total energy difference and the inter-ionic displacement should be less than 5 × 10^−5^ eV/atom and 0.005 Å/atom, respectively. We first established the 3 × 3 × 1 supercell of cubic of FeCoSn(OH)_6_ with Co and Fe occupying the same Co sites, which shows a ratio of 3:1. This structure composition is consistent with experiment characterizations. To construct the amorphous structure, the MD simulations have been performed on FeCoSn(OH)_6_ under the NVT conditions at 573 K to be consistent with the experimental condition. The time step was set as 1 fs and the total simulation time is 5 ps with 5000 simulation steps to obtain the amorphous structure. As the simulation time increases, the structure starts to become amorphous. After the simulation finishes, we further carried out the geometry optimizations to obtain the stabilized amorphous structure as the FeCoSn(OH)_6_-300. We have cleaved four layers of the amorphous structure from the (001) facet. Meanwhile, to understand the amorphous transformation temperature, the MD simulations have been carried out under different temperatures from 298 to 673 K under the NVT conditions. The time step is 1 fs and the total simulation time is 5 ps with 5000 simulation steps.

## Supplementary information

Supplementary Information

## Data Availability

The data generated in this study are provided in Source Data file. Source data are provided with this paper.

## Source data

Source Data
