# Supplementary material for: A top-down strategy for amorphization of hydroxyl compounds for electrocatalytic oxygen evolution
Source: Nat Commun. 2022 Mar 4;13:1187. doi: 10.1038/s41467-022-28888-3 (PMC8897429; doi:10.1038/s41467-022-28888-3)
Supplement: Supplementary file 1 — Supplementary Information [file 41467_2022_28888_MOESM1_ESM.pdf]

Supporting Information

A top-down strategy for amorphization of hydroxyl compounds for electrocatalytic oxygen evolution

Shangheng Liu[1,2], Shize Geng[2], Ling Li[2], Ying Zhang[1], Guomian Ren[3], Bolong Huang[4,*], Zhiwei Hu[5], Jyh-Fu Lee[6], Yu-Hong Lai[7], Ying-Hao Chu[7], Yong Xu[3,*], Qi Shao[2] and Xiaoqing Huang[1,*]

[1]State Key Laboratory of Physical Chemistry of Solid Surfaces, College of Chemistry and Chemical Engineering, Xiamen University, Xiamen, 361005, China.

[2]College of Chemistry, Chemical Engineering and Materials Science, Soochow University, Jiangsu, 215123, China.

[3]Guangzhou Key Laboratory of Low-Dimensional Materials and Energy Storage Devices, Collaborative Innovation Center of Advanced Energy Materials, School of Materials and Energy, Guangdong University of Technology, Guangzhou, 510006, China.

[4]Department of Applied Biology and Chemical Technology, The Hong Kong Polytechnic University, Hung Hom, Kowloon, Hong Kong SAR, China.

[5]Max Planck Institute for Chemical Physics of Solids, Nothnitzer Strasse 40, Dresden, 01187, Germany.

[6]National Synchrotron Radiation Research Center, 101 Hsin-Ann Road, Hsinchu, 30076, Taiwan.

[7]Department of Materials Science and Engineering, National Yang Ming Chiao Tung University, Hsinchu, 30010, Taiwan.

*To whom correspondence should be addressed.

E-mail: bhuang@polyu.edu.hk, yongxu@gdut.edu.cn, hxq006@xmu.edu.cn

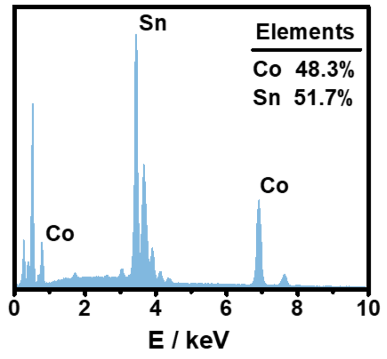

Supplementary Figure 1. EDS pattern of the pristine $CoSn(OH)_6$.

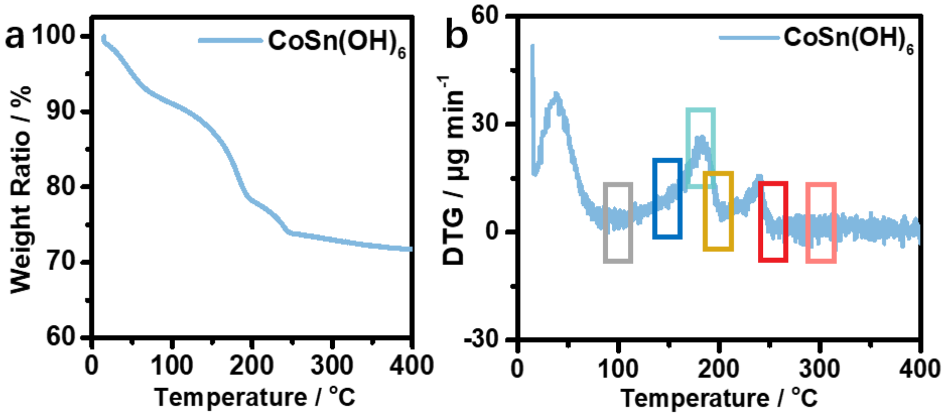

Supplementary Figure 2. (**a**) TGA and (**b**) DTG curves of $CoSn(OH)_6$.

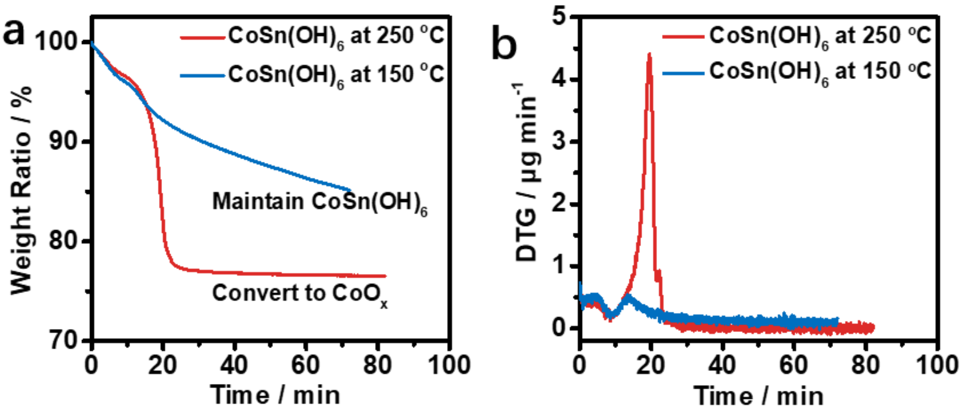

Supplementary Figure 3. (**a**) TGA and (**b**) DTG curves of $CoSn(OH)_6$ collected at 150 and 250 °C.

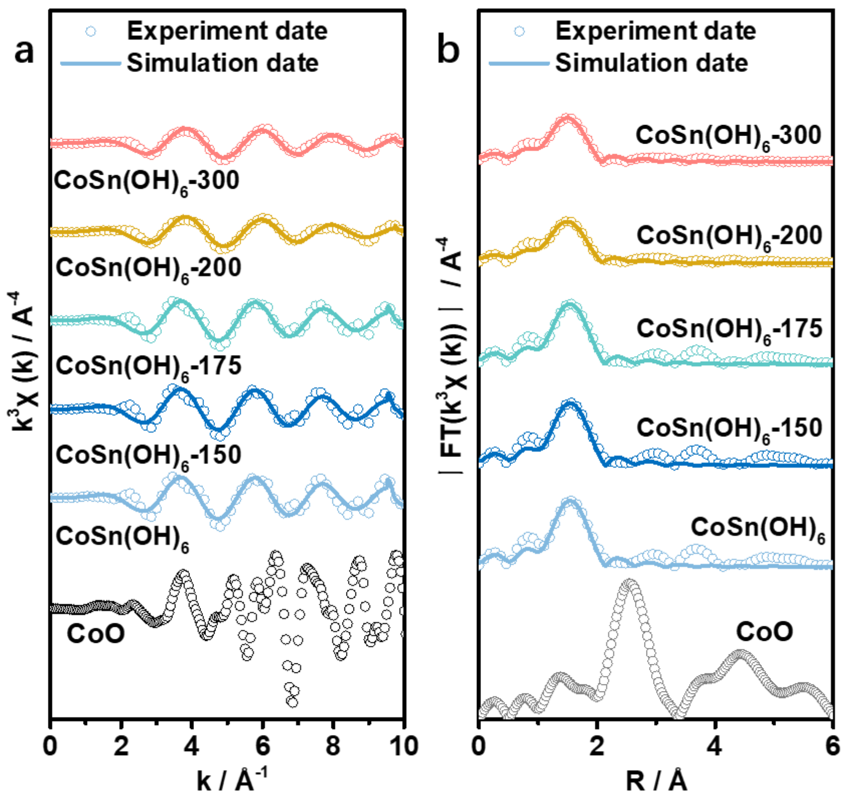

Supplementary Figure 4. The fitting EXAFS spectra at Co *K*-edge (**a**) *K* space and (**b**) *R* space for CoO, $CoSn(OH)_6$, $CoSn(OH)_6$-150, $CoSn(OH)_6$-175, $CoSn(OH)_6$-200 and $CoSn(OH)_6$-300.

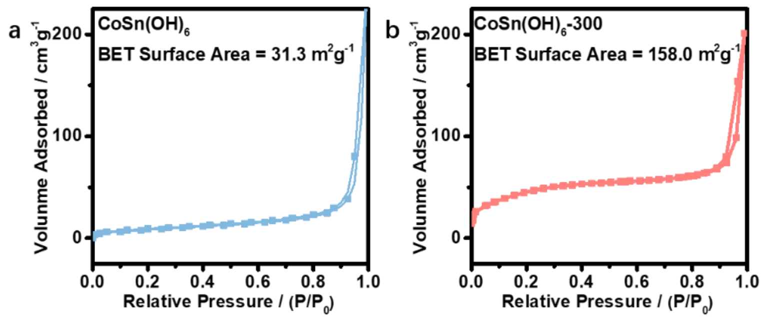

Supplementary Figure 5. The N_2 adsorption-desorption isotherms of (**a**) $CoSn(OH)_6$ and (**b**) $CoSn(OH)_6$-300.

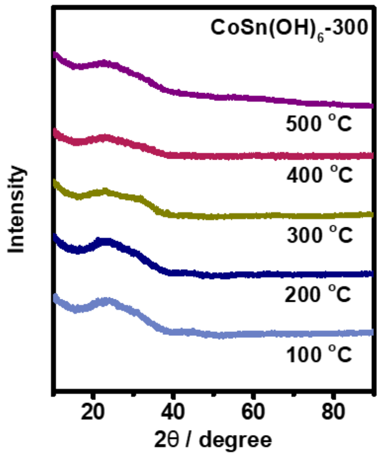

Supplementary Figure 6. XRD patterns of $CoSn(OH)_6$-300 treated at different temperatures.

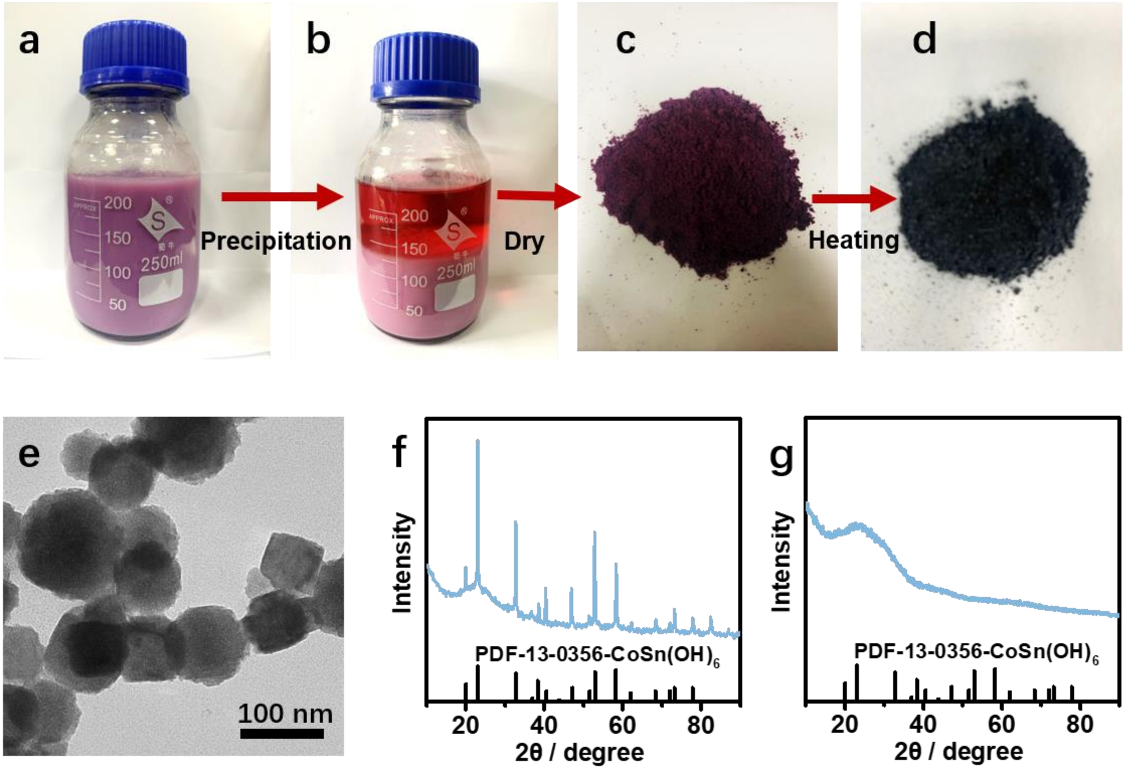

Supplementary Figure 7. Pictures of the scaled-up synthesis of $CoSn(OH)_6$ at (**a**) initial and (**b**) final stage. Pictures of (**c**) $CoSn(OH)_6$ and (**d**) $CoSn(OH)_6$-300. (**e**) TEM image of $CoSn(OH)_6$. XRD patterns of (**f**) $CoSn(OH)_6$ and (**g**) $CoSn(OH)_6$-300.

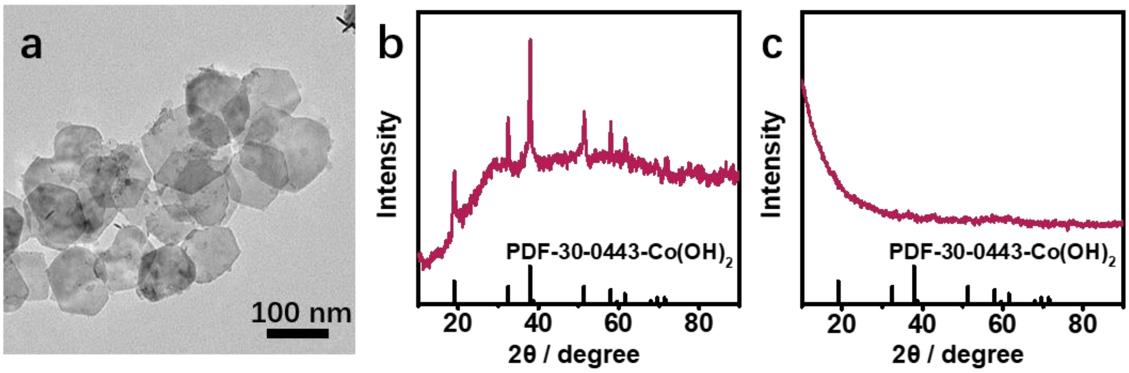

Supplementary Figure 8. (**a**) TEM image of $Co(OH)_2$. XRD patterns of (**b**) $Co(OH)_2$ and (**c**) $Co(OH)_2$-400. $Co(OH)_2$-400 was obtained by treating $Co(OH)_2$ in Ar at 400 ^oC for 1 h.

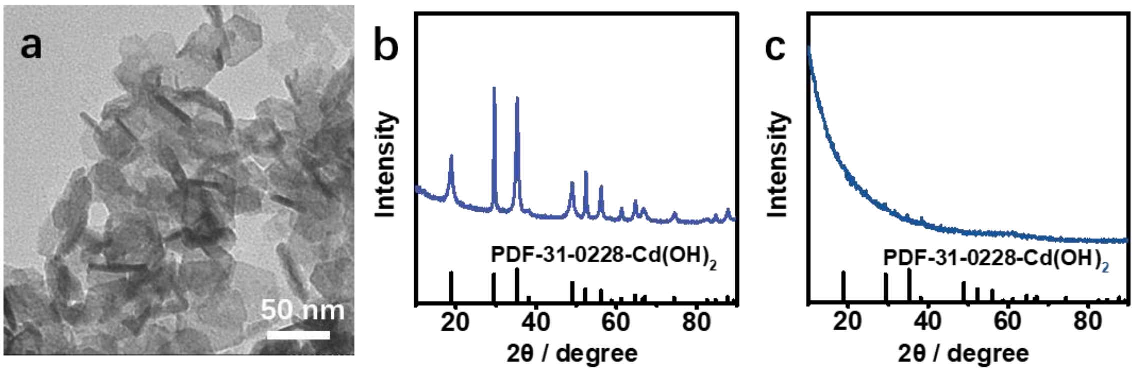

Supplementary Figure 9. (**a**) TEM image of $Cd(OH)_2$. XRD patterns of (**b**) $Cd(OH)_2$ and (**c**) $Cd(OH)_2$-400. $Cd(OH)_2$-400 was obtained by treating $Cd(OH)_2$ in Ar at 400 ^{o}C for 1 h.

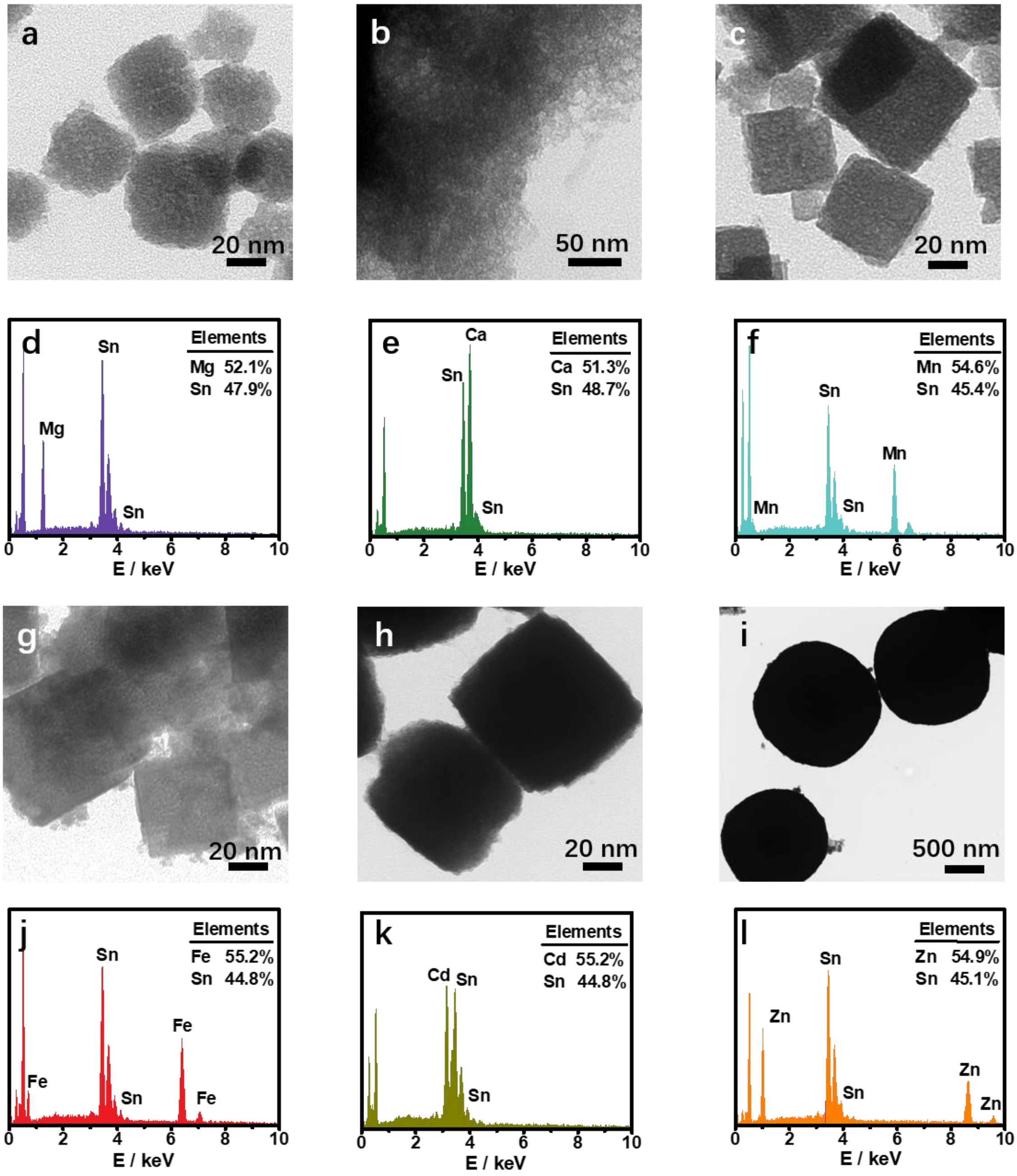

Supplementary Figure 10. (**a, b, c, g, h, i**) TEM images and (**d, e, f, j, k, l**) EDS patterns of (**a, d**) $MgSn(OH)_6$, (**b, e**) $CaSn(OH)_6$, (**c, f**) $MnSn(OH)_6$, (**g, j**) $FeSn(OH)_6$, (**h, k**) $ZnSn(OH)_6$ and (**i, l**) $CdSn(OH)_6$.

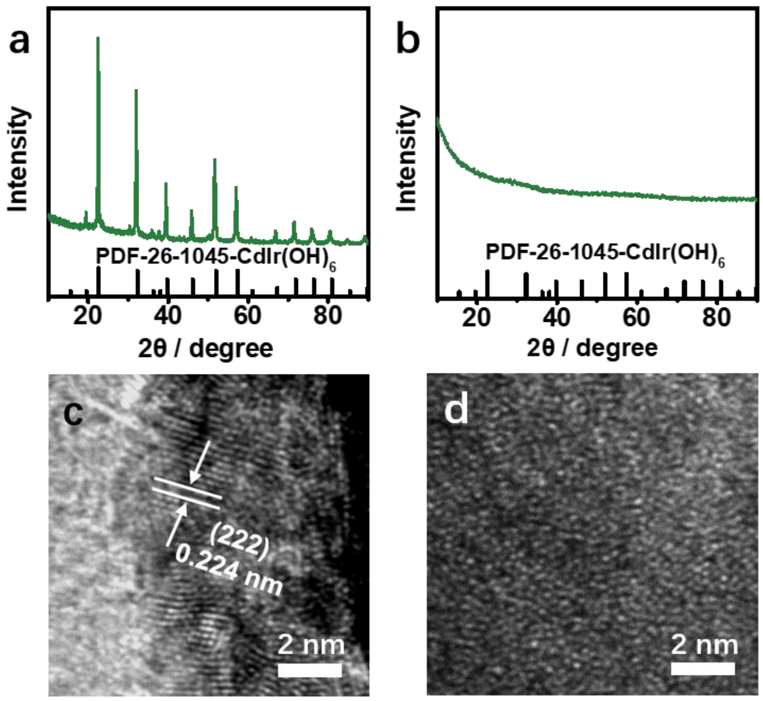

Supplementary Figure 11. XRD patterns of (**a**) $CdIr(OH)_6$ and (**b**) $CdIr(OH)_6$-300. HRTEM images of (**c**) $CdIr(OH)_6$ and (**d**) $CdIr(OH)_6$-300. $CdIr(OH)_6$-300 was obtained by treating $CdIr(OH)_6$ in Ar at 300 ^oC for 1 h.

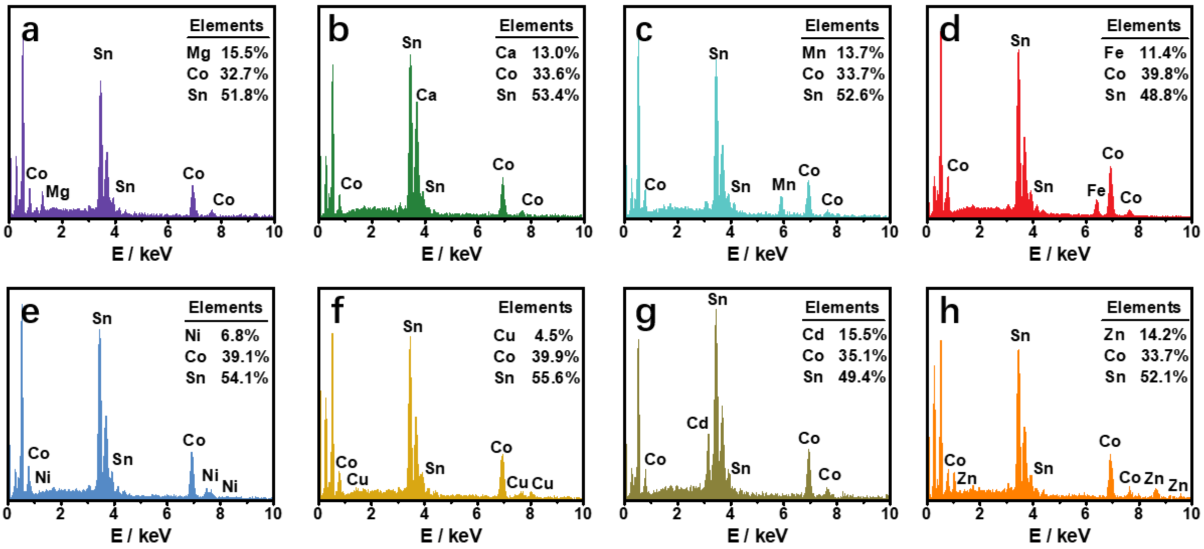

Supplementary Figure 12. EDS patterns of (**a**) $MgCoSn(OH)_6$, (**b**) $CaCoSn(OH)_6$, (**c**) $MnCoSn(OH)_6$, (**d**) $FeCoSn(OH)_6$, (**e**) $NiCoSn(OH)_6$, (**f**) $CuCoSn(OH)_6$, (**g**) $CdCoSn(OH)_6$ and (**h**) $ZnCoSn(OH)_6$.

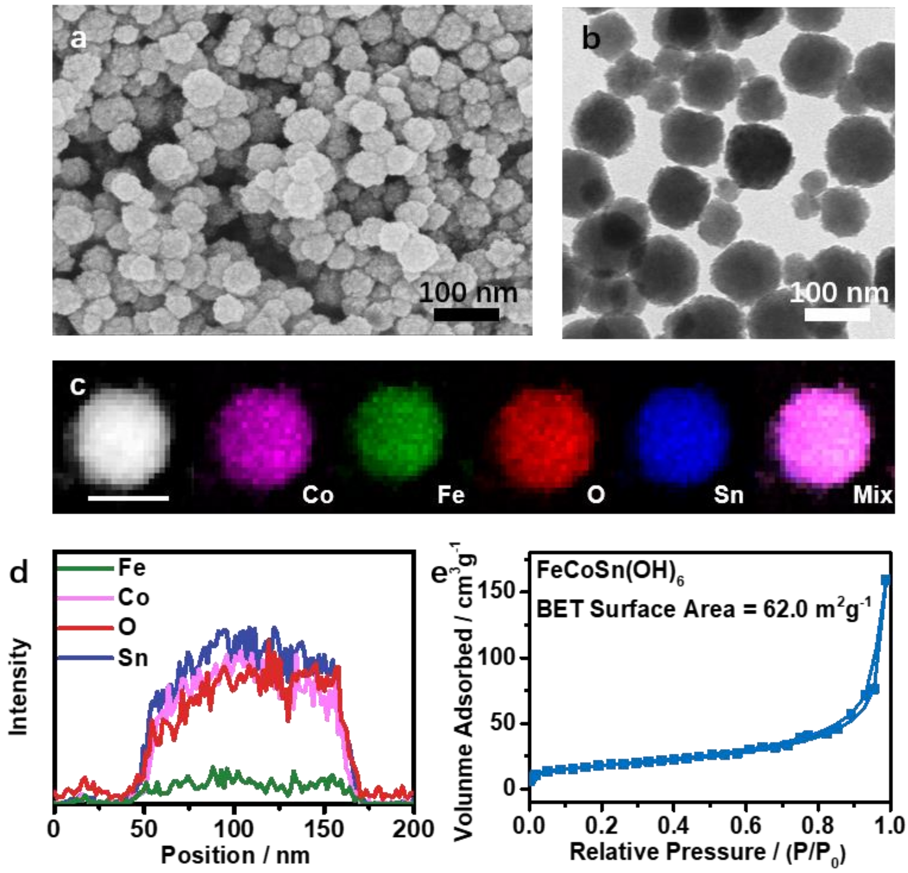

Supplementary Figure 13. (**a**) SEM image, (**b**) TEM image, (**c**) HAADF-STEM image and corresponding elemental mapping images, (**d**) Line-scan analysis and (**e**) N_2 adsorption-desorption isotherms of $FeCoSn(OH)_6$. Scale bar in (**c**) is 100 nm.

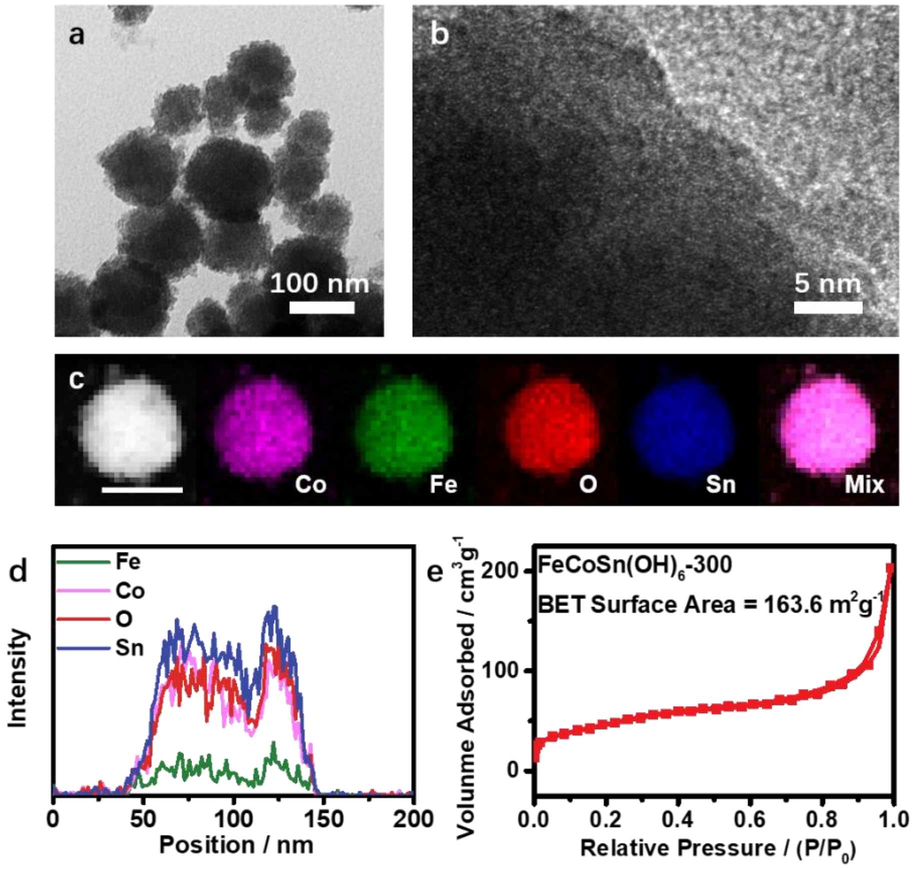

Supplementary Figure 14. (**a**) TEM image, (**b**) HRTEM image, (**c**) HAADF-STEM image and corresponding elemental mappings, (**d**) Line-scan analysis and (**e**) N_2 adsorption-desorption isotherms of $FeCoSn(OH)_6$-300. Scale bar in (**c**) is 100 nm. $FeCoSn(OH)_6$-300 was obtained by treating $FeCoSn(OH)_6$ in Ar at 300 °C for 1 h.

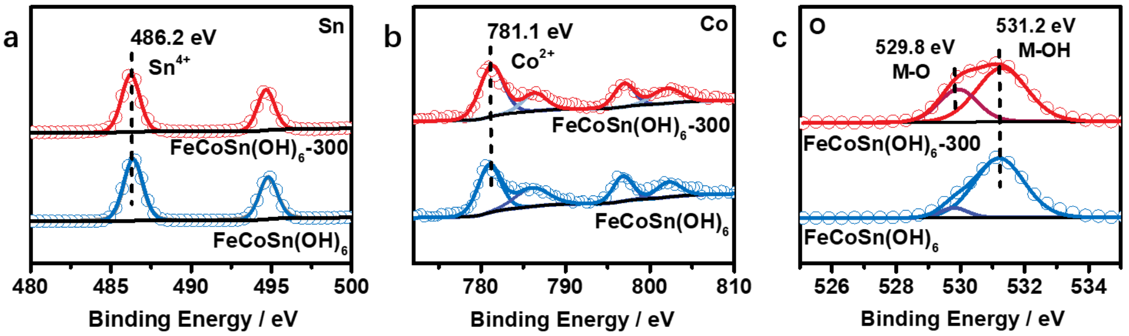

Supplementary Figure 15. (**a**) Sn 3*d*, (**b**) Co 2*p* and (**c**) O 1*s* XPS spectra of $FeCoSn(OH)_6$ and $FeCoSn(OH)_6$-300.

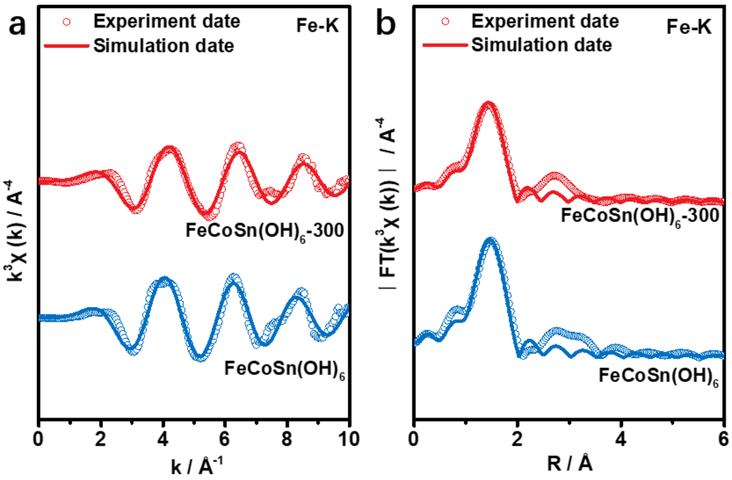

Supplementary Figure 16. Fe *K*-edge EXAFS fitting curves at (**a**) *K* and (**b**) *R* space for $FeCoSn(OH)_6$ and $FeCoSn(OH)_6$-300.

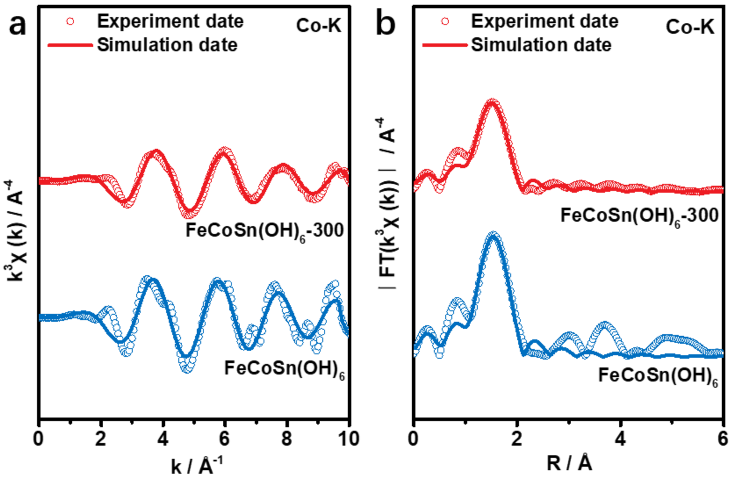

Supplementary Figure 17. Co *K*-edge EXAFS fitting curves at (**a**) *K* and (**b**) *R* space for $FeCoSn(OH)_6$ and $FeCoSn(OH)_6$-300.

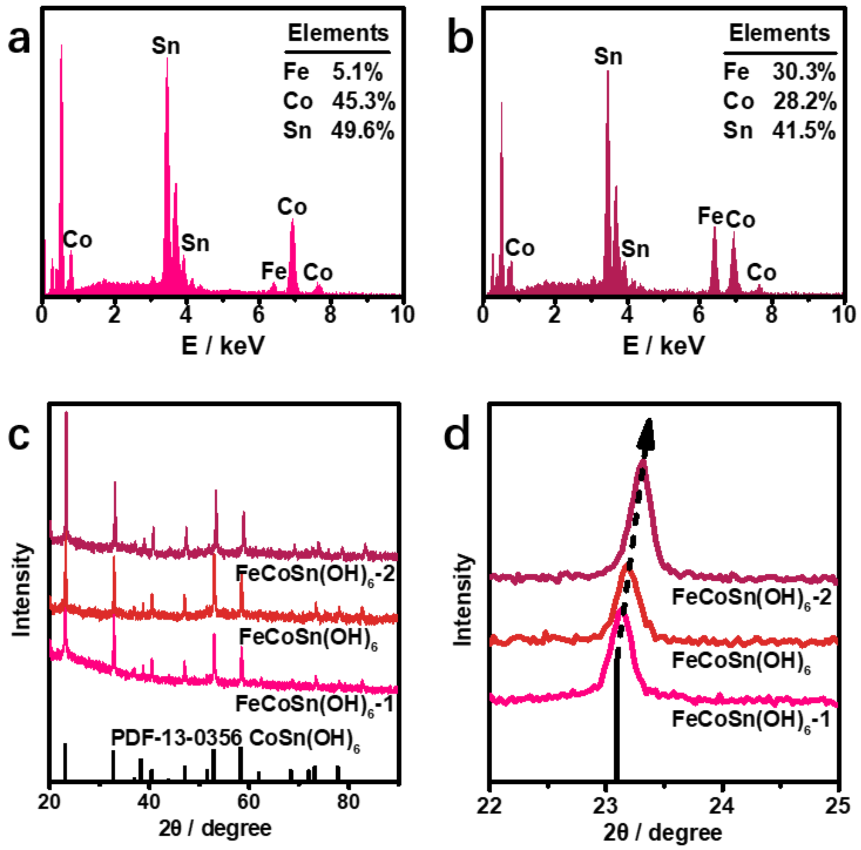

Supplementary Figure 18. The EDS patterns of (**a**) $FeCoSn(OH)_6$-1 ($Fe_{0.1}Co_{0.9}Sn(OH)_6$) and (**b**) $FeCoSn(OH)_6$-2 ($Fe_{0.5}Co_{0.5}Sn(OH)_6$). (**c**) The XRD pattern. (**d**) The enlarged XRD pattern at 22-25°.

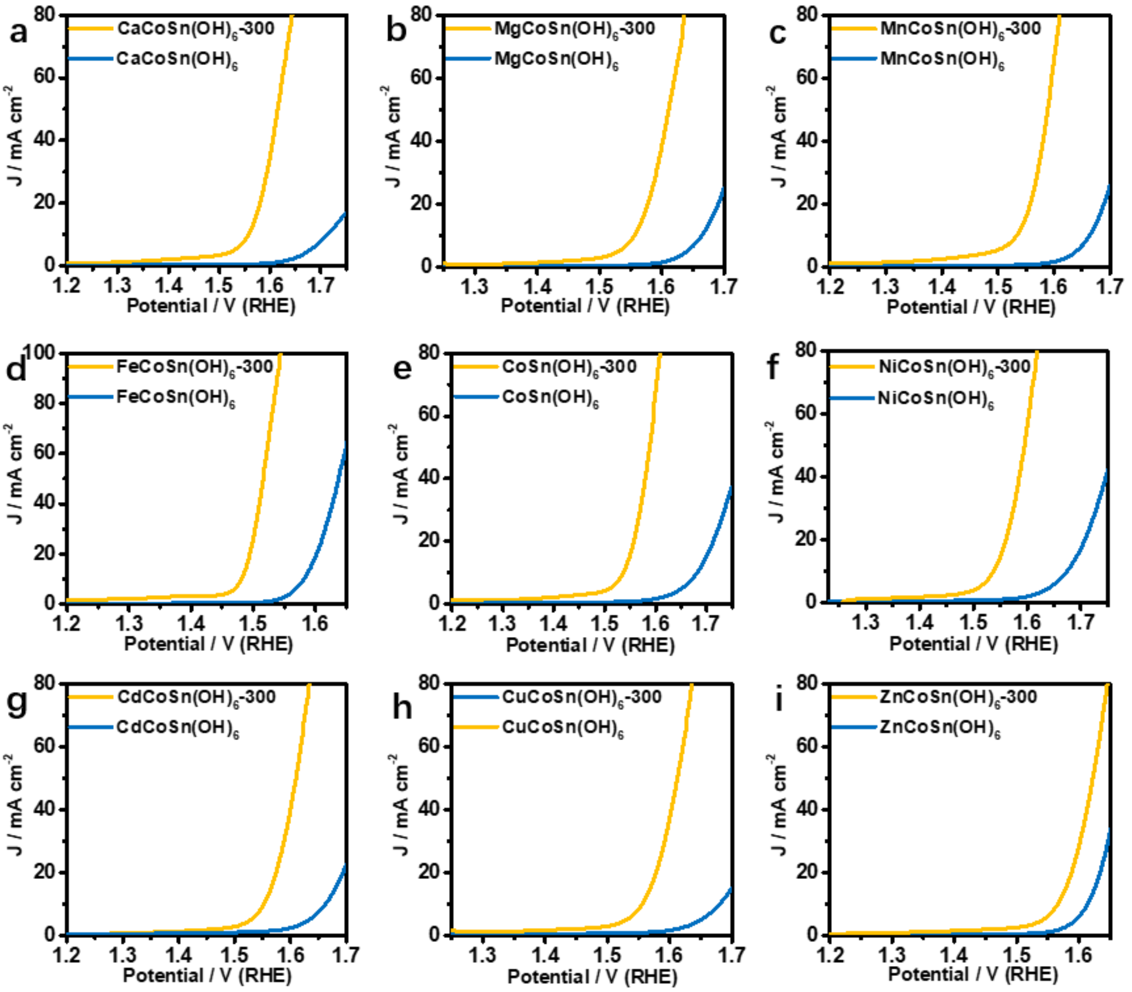

Supplementary Figure 19. OER polarization curves of amorphous $MCoSn(OH)_6$-300 and crystalline $MCoSn(OH)_6$. (**a**) $CaCoSn(OH)_6$-300 and $CaCoSn(OH)_6$, (**b**) $MgCoSn(OH)_6$-300 and $MgCoSn(OH)_6$, (**c**) $MnCoSn(OH)_6$-300 and $MnCoSn(OH)_6$, (**d**) $FeCoSn(OH)_6$-300 and $FeCoSn(OH)_6$, (**e**) $CoSn(OH)_6$-300 and $CoSn(OH)_6$, (**f**) $NiCoSn(OH)_6$-300 and $NiCoSn(OH)_6$, (**g**) $CdCoSn(OH)_6$-300 and $CdCoSn(OH)_6$, (**h**) $CuCoSn(OH)_6$-300 and $CuCoSn(OH)_6$, (**i**) $ZnCoSn(OH)_6$-300 and $ZnCoSn(OH)_6$.

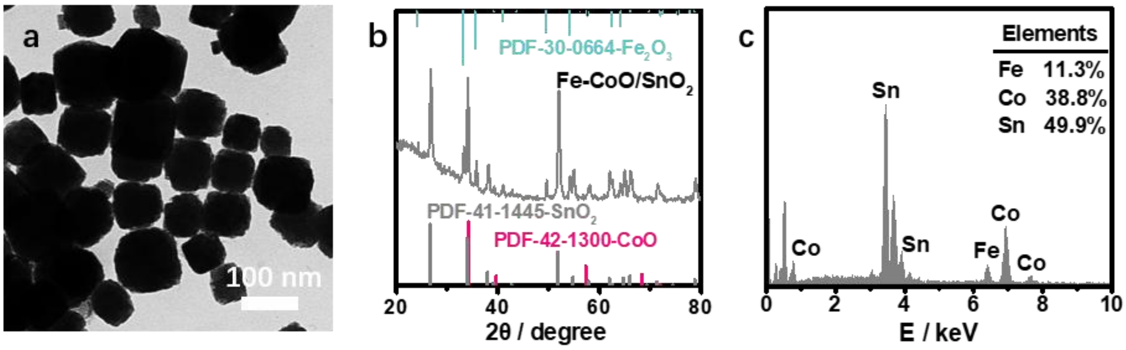

Supplementary Figure 20. (**a**) TEM image, (**b**) XRD pattern, (**c**) SEM-EDS patternof Fe-CoO/SnO_2.

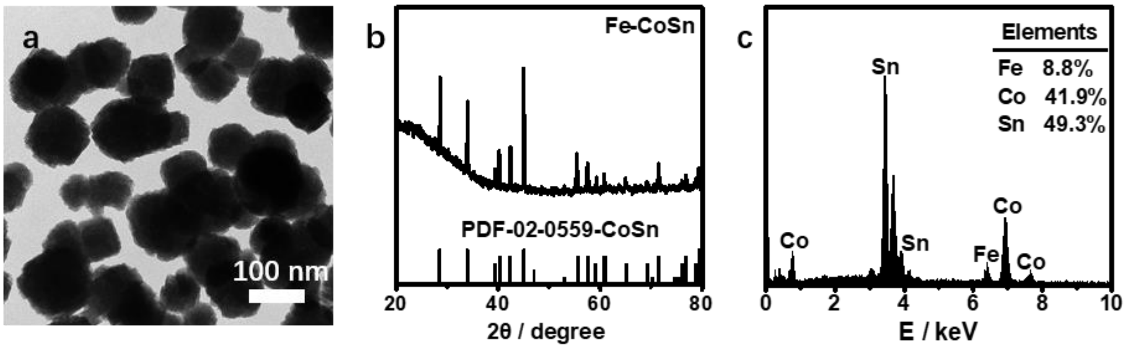

Supplementary Figure 21. (**a**) TEM image, (**b**) XRD pattern, (**c**) SEM-EDS pattern of Fe-CoSn.

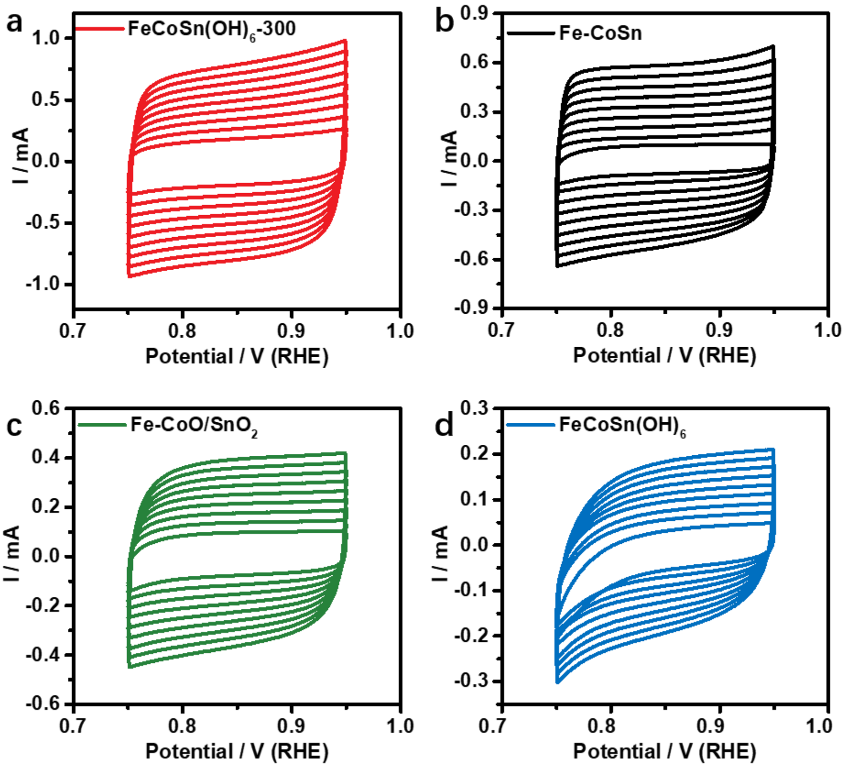

Supplementary Figure 22. CV curves collected at the range of 0.75 to 0.95 V vs. RHE with scan rate from 40 to 200 mV s^{-1} of (**a**) $FeCoSn(OH)_6$-300, (**b**) Fe-CoSn, (**c**) Fe-CoO/SnO_2 and (**d**) $FeCoSn(OH)_6$.

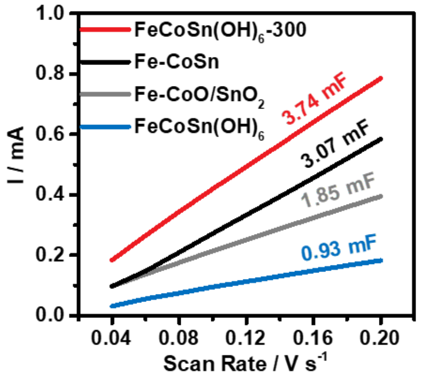

Supplementary Figure 23. Electrochemical double-layer capacitance (C_{dl}) of $FeCoSn(OH)_6$-300, Fe-CoSn, Fe-CoO/SnO_2 and $FeCoSn(OH)_6$.

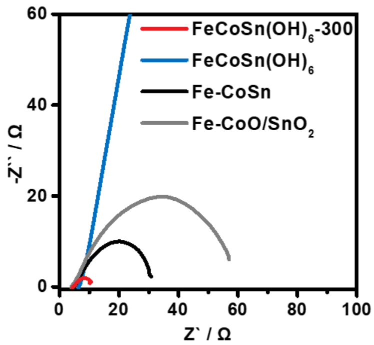

Supplementary Figure 24. EIS of $FeCoSn(OH)_6$-300, $FeCoSn(OH)_6$, Fe-CoSn and Fe-CoO/SnO_2.

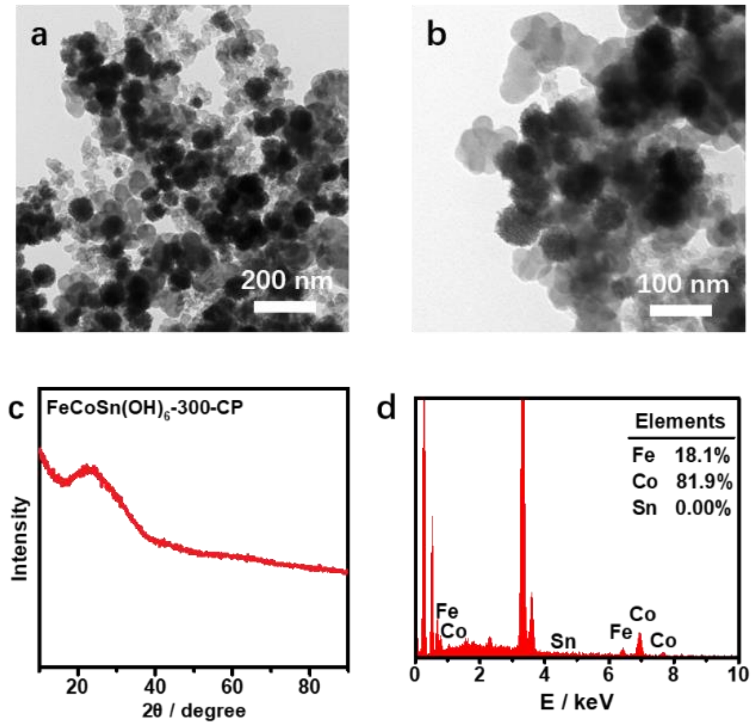

Supplementary Figure 25. (**a, b**) TEM images, (**c**) XRD pattern and (**d**) EDS of $FeCoSn(OH)_6$-300 after OER.

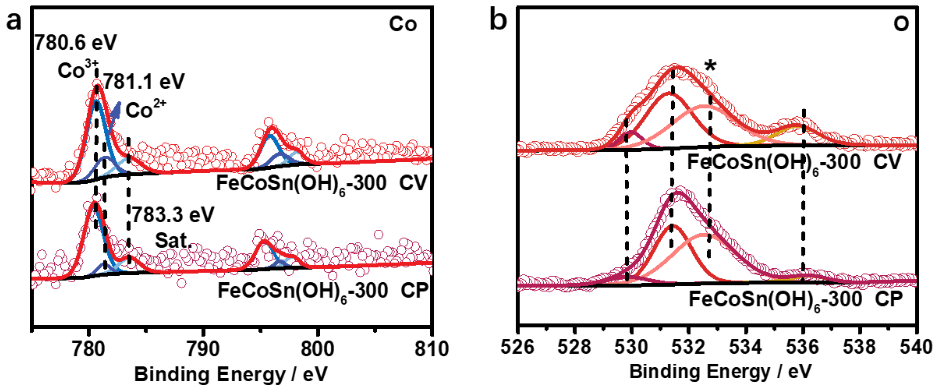

Supplementary Figure 26. (**a**) Co 2*p* and (**b**) O 1*s* XPS spectra of $FeCoSn(OH)_6$-300 CV (after electrochemical activation) and $FeCoSn(OH)_6$-300 CP (after stability testing). * in (b) represents as CoOOH.

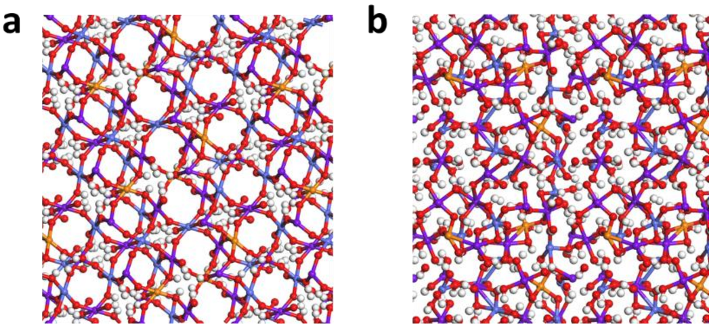

Supplementary Figure 27. The models of (a) $FeCoSn(OH)_6$ and (b) $FeCoSn(OH)_6$-300. Orange balls = Fe, blue balls = Co, purple balls = Sn, red balls = O and white balls = H.

Supplementary Table 1. EXAFS fitting results using the ARTEMIS module of IFEFFIT.

Sample	Path	CN	R / Å	σ^2 / 10^{-3}Å^2	ΔE_0 / eV	R factor
$CoSn(OH)_6$-300	Co-O	4.2	2.02±0.02	7.9±2.5	-5.6±2.2	0.0073
$CoSn(OH)_6$-200	Co-O	4.5	2.02±0.02	9.8±3.5	-5.1±2.8	0.0122
$CoSn(OH)_6$-175	Co-OH	5.7	2.08±0.03	7.5±4.1	-4.7±3.3	0.0187
$CoSn(OH)_6$-150	Co-OH	5.8	2.08±0.03	7.4±4.0	-4.4±3.3	0.0192
$CoSn(OH)_6$	Co-OH	6	2.08±0.03	6.4±3.9	-4.5±3.3	0.0182

Supplementary Table 2. EXAFS fitting results using the ARTEMIS module of IFEFFIT.

Sample	Path	CN	R / Å	σ^2 / 10^{-3}Å^2	ΔE_0 / eV	R factor
$FeCoSn(OH)_6$	Fe-OH	5.0	1.96±0.02	7.4±3.8	-4.5±3.5	0.0174
$FeCoSn(OH)_6$-300	Fe-O	3.9	1.92±0.03	7.0±3.9	-4.7±4.1	0.0217
$FeCoSn(OH)_6$	Co-OH	5.6	2.07±0.03	7.0±3.9	-5.0±3.3	0.0185
$FeCoSn(OH)_6$-300	Co-O	4.4	2.04±0.02	8.3±3.0	-5.1±2.7	0.0110

Supplementary Table 3. Comparison between $FeCoSn(OH)_6$-300 and other reported FeCoNi-based catalysts for OER in alkaline electrolyte.

Catalyst	Electrolyte	Overpotential/ mV	Tafel slope/ mV dec^{-1}	**Ref.**
$FeCoSn(OH)_6$-300	1 M KOH	**266**	**39.3**	**This work**
G-FeCoW	1 M KOH	223		*Science* **352**, 333–337 (2016)
A-FeCoW	1 M KOH	301		*Science* **352**, 333–337 (2016)
FeCoMoW on carbon paper	1 M KOH	212		*Nat. Catal.* **3**, 985–992 (2020)
Co-Fe-N-C	1 M KOH	320	40	*Nat. Energy* **6**, 1054–1066 (2021)
$Zn_{0.2}Co_{0.8}OOH$	1 M KOH	235	35.7	*Nat. Energy* **4**, 329–338 (2019)
WC_x-FeNi	1 M KOH	237	44	*Nat. Mater.* **20**, 1240–1247 (2021)
CoFe LDH	1 M KOH	404		*Nat. Commun.* **11**, 2522 (2020)
Co-N-C	1 M KOH	321	40	*J. Am. Chem. Soc.* **141**, 14190–14199 (2019)
$NiCo_{2-x}Fe_xO_4$ NBs	1 M KOH	274	42	*Angew. Chem. Int. Ed.* **60**, 11841–11846 (2021)
Fe-UNT	1 M KOH	270	36.6	*Angew.Chem. Int.Ed.* **59**, 2313–2317 (2020)
CoO/Co_3O_4 on Ti foil	1 M KOH	270	55	*Angew. Chem. Int. Ed.* **59**, 6929–6935 (2020)
Ni-Co-P nanoboxes	1 M KOH	330	96	*Angew. Chem., Int. Ed.* **56**, 3897 (2017)
CoO_x NPs/BNG	0.1 M KOH	295	57	*Angew. Chem. Int. Ed.* **56**, 7121 (2017)
A-$CoS_{4.6}O_{0.6}$ PNCs	1 M KOH	290	67	*Angew. Chem. Int. Ed.* **56**, 4858 (2017)
Co_3O_4/Co-Fe oxide	1 M KOH	297	61	*Adv. Mater.* **30**, 1801211 (2018)
Co(OH)F	1 M KOH	313	52.8	*Adv. Mater.* **29**, 1700286 (2017)
Fe-Co-2.3Ni-B	1 M KOH	274	38	*Adv. Energy Mater.* **8**, 1701475 (2018)
Fe_3C-Co	1 M KOH	340		*Adv. Funct. Mater.* **29**, 1901949 (2019)
$Fe_5Co_4Ni_{20}Se_{36}B_x$	1 M KOH	279.8	59.5	*ACS Nano* **13**, 11469−11476 (2019)
rGO@$CoFe_2O_4$	1 M KOH	300	36	*ACS Catal.* **9**, 3878-3887 (2019)

Supplementary Table 4. Parameters for the synthesis of binary and ternary hydroxides.

Sample	Precursors (M)	Precursors (Co)	Precursors (Sn)
$CoSn(OH)_6$	-	$CoCl_2$ (0.2 mmol)	$SnCl_4·5H_2O$ (0.2 mmol)
$MgCoSn(OH)_6$	$MgCl_2$ (0.04 mmol)	$CoCl_2$ (0.16 mmol)	$SnCl_4·5H_2O$ (0.2 mmol)
$CaCoSn(OH)_6$	$CaCl_2$ (0.04 mmol)	$CoCl_2$ (0.16 mmol)	$SnCl_4·5H_2O$ (0.2 mmol)
$MnCoSn(OH)_6$	$MnCl_2$ (0.04 mmol)	$CoCl_2$ (0.16 mmol)	$SnCl_4·5H_2O$ (0.2 mmol)
$FeCoSn(OH)_6$	$FeCl_2$ (0.04 mmol)	$CoCl_2$ (0.16 mmol)	$SnCl_4·5H_2O$ (0.2 mmol)
$NiCoSn(OH)_6$	$NiCl_2$ (0.04 mmol)	$CoCl_2$ (0.16 mmol)	$SnCl_4·5H_2O$ (0.2 mmol)
$CuCoSn(OH)_6$	$CuCl_2$ (0.04 mmol)	$CoCl_2$ (0.16 mmol)	$SnCl_4·5H_2O$ (0.2 mmol)
$CdCoSn(OH)_6$	$CdCl_2$ (0.04 mmol)	$CoCl_2$ (0.16 mmol)	$SnCl_4·5H_2O$ (0.2 mmol)
$ZnCoSn(OH)_6$	$ZnCl_2$ (0.04 mmol)	$CoCl_2$ (0.16 mmol)	$SnCl_4·5H_2O$ (0.2 mmol)
